# Humoral Immunity Profiling to Pandemic and Bat‐Derived Coronavirus Variants: A Geographical Comparison

**DOI:** 10.1002/advs.202403503

**Published:** 2024-10-29

**Authors:** Parinaz Fathi, Andrea Lucia Alfonso, Christina Yek, Zoe Putman, Matthew Drew, Dominic Esposito, Irfan Zaidi, Sophana Chea, Sokna Ly, Rathanak Sath, Chanthap Lon, Huch Chea, Rithea Leang, Rekol Huy, Sovann Ly, Heng Seng, Chee Wah Tan, Feng Zhu, Lin‐Fa Wang, Fabiano Oliveira, Kaitlyn Sadtler, Jessica Manning

**Affiliations:** ^1^ Section on Immunoengineering Biomedical Engineering and Technology Acceleration Center National Institute of Biomedical Imaging and Bioengineering Bethesda MD 20892 USA; ^2^ Laboratory of Malaria and Vector Research National Institute of Allergy and Infectious Diseases Rockville MD 20892 USA; ^3^ Protein Expression Laboratory NCI RAS Initiative Frederick National Laboratory for Cancer Research Frederick MD 21701 USA; ^4^ Laboratory of Malaria Immunology and Vaccinology National Institute of Allergy and Infectious Diseases Bethesda MD 20892 USA; ^5^ International Center of Excellence in Research Cambodia National Institute of Allergy and Infectious Diseases Phnom Penh 120801 Cambodia; ^6^ National Center for Parasitology, Entomology, and Malaria Control Ministry of Health Phnom Penh 120801 Cambodia; ^7^ Cambodian Center for Disease Control Ministry of Health Phnom Penh 120407 Cambodia; ^8^ Programme for Emerging Infectious Diseases Duke‐National University of Singapore Medical School 169857 Singapore Singapore; ^9^ Infectious Diseases Translational Research Programme Department of Microbiology and Immunology Yong Loo Lin School of Medicine National University of Singapore 117597 Singapore Singapore; ^10^ Present address: Sanofi Washington DC 20004 USA

**Keywords:** antibodies, bats, betacoronaviruses, cross‐reactivity, SARS‐CoV‐2

## Abstract

Dynamic pathogen exposure may impact the immunological response to SARS‐CoV‐2 (SCV2). One potential explanation for the lack of severe SCV2‐related morbidity and mortality in Southeast Asia is prior exposure to related betacoronaviruses. Recent discoveries of SCV2‐related betacoronaviruses from horseshoe bats (*Rhinolophus sinicus*) in Thailand, Laos, and Cambodia suggest the potential for bat‐to‐human spillover exposures in the region. In this work, serum antibodies to protein constructs from SCV2 and a representative bat coronavirus isolated in Cambodia (RshSTT182) are measured in pre‐pandemic Cambodian human sera using ELISA assays. Of 293 Cambodian samples tested (*N* = 131 with acute malaria, *n* = 162 with acute undifferentiated febrile illness), 32 (10.9%) are seropositive for SCV2 based on established Spike and receptor‐binding domain (RBD) cutoffs. Within SCV2 seropositive samples, 16 (50%) have higher antibody levels to antigens from the representative virus RshSTT182 versus SCV2 antigens; competitive binding ELISA assays demonstrate inhibition of reactivity to SCV2 Spike after pre‐incubation with RshSTT182 Spike. Surrogate virus neutralization tests demonstrate that 8/30 (26.7%) SCV2 ELISA positive pre‐pandemic Cambodian samples have neutralizing activity against SCV2, while 14/30 (46.7%) have activity against other SCV2‐related betacoronaviruses. These data suggest that exposure to related betacoronaviruses may elicit cross‐reactive immunity to SCV2 prior to the global pandemic.

## Graphical abstract

Understanding the role of geographic exposure to coronaviruses in the development of cross‐reactive immunity is important for pandemic modeling and interpretation of serology. In this work, the reactivity of U.S. pandemic samples, pre‐pandemic Cambodian samples, and pre‐pandemic U.S. samples is evaluated to SARS‐CoV‐2 (SCV2) and other coronaviruses. Cross‐reactivity is observed between SCV2 and bat‐derived viruses.

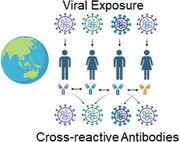

## 1 Introduction

Antibodies produced as part of humoral immune responses play an important role in combating viral infections, including infection by SARS‐CoV‐2 (SCV2).^[^ ^1^, ^2^ ^]^ Many studies have explored SCV2 pathogenesis and associated immune responses.^[^ ^3^, ^4^, ^5^, ^6^, ^7^, ^8^, ^9^, ^10^ ^]^ The existence of antibodies that have cross‐reactivity to SCV2 antigens raises the question of whether prior geographic exposure to other viruses can lead to the development of antibodies that cross‐react with SCV2.

Horseshoe bats (*Rhinolophus spp*.) are known hosts for a variety of viruses belonging to the *Sarbecovirus* subgenus of betacoronaviruses (bCoV).^[^ ^11^ ^]^ The impact of bat exposure on the breadth and function of bCoV‐specific antibodies could be one explanation for higher‐than‐expected serological reactivity to SCV2 in Southeast Asia prior to the pandemic.^[^ ^12^, ^13^ ^]^ Indeed, prior SARS‐CoV‐1 (SCV1) infection in humans immunized with a SCV2 vaccine resulted in cross‐clade boosting of neutralizing antibodies against pan‐sarbecoviruses.^[^ ^14^ ^]^ Recent surveys of *Rhinolophus* individuals from Thailand, Laos, and Cambodia^[^ ^15^, ^16^ ^]^ have led to discoveries of novel bCoV, including several viruses highly homologous to SCV2. A more recent report described the discovery of several bCoVs isolated from Laotian *Rhinolophus* species and confirmed the virus’ ability to bind hACE2.

In Cambodia and other parts of the Greater Mekong Subregion that border China, bat guano farming, via the construction of +artificial bat roosting near personal domiciles to collect nutrient‐rich fecal droppings, is a lucrative enterprise.^[^ ^17^, ^18^ ^]^ Notably, the guano collection process is typically performed without personal protective equipment and is in the same area where persons, children, and domestic animals co‐reside. Undetected zoonotic viral transmission from bats to humans could plausibly occur in these settings.

A counter‐explanation for the higher levels of pre‐pandemic serological activity to SCV2 in some areas of the world is the cross‐reactivity of antibodies from *Plasmodium spp*. Infection.^[^ ^12^, ^19^ ^]^ Examples from individuals living in Africa and Southeast Asia reveal higher‐than‐expected total Immunoglobulin G (IgG) levels to SCV2 Spike, although these antibodies are largely non‐functional. The dysregulated polyclonal B cell expansion that occurs in acute malaria may raise cross‐reactive antibodies to SCV2.^[^ ^13^ ^]^ Specifically, it is hypothesized that a sialic moiety at the N‐terminus of SCV2 Spike protein interacts with *Plasmodium falciparum* antibodies, as signals are abolished when antibodies are incubated with desialylated S1 subunits. Taken together, there are clearly delineated, country‐specific pathogen exposure profiles that drive serological reactivity to SCV2 Spike and receptor‐binding domain (RBD) proteins.

In order to further understand the higher rates of pre‐pandemic seroreactivity in the Cambodian population, we characterized the humoral immunity of rural and peri‐urban Cambodians to Spike, RBDs, and N‐terminal domains (NTDs) of SCV2 against healthy pre‐pandemic U.S. sera, as well as convalescent U.S. patient samples from New York City. To expand our findings, we tested both sialylated and desialylated proteins for wild‐type SCV2 RBD and NTD, SCV2 variants including B.1.1.7, B.1.1.529, B.1.617.2, and E484K strains, and whole Spike, RBD, NTD, desialylated RBD, and desialylated NTD proteins of RshSTT182, a bat‐borne bCoV (“BatCoV”) isolated from a Cambodian bat in 2010 (Table S1, Supporting Information).^[^ ^20^ ^]^ We found that pre‐pandemic Cambodian samples had reactivity to SCV2 and variant antigens that could not entirely be explained by *Plasmodium spp*. Infection. Additionally, some Cambodian samples exhibited neutralizing activity against SCV2 and bCoV antigens.

## 2 Results

### 2.1 Pre‐Pandemic Cambodian Samples Exhibit SCV2 Seroreactivity

**Figure 1:**
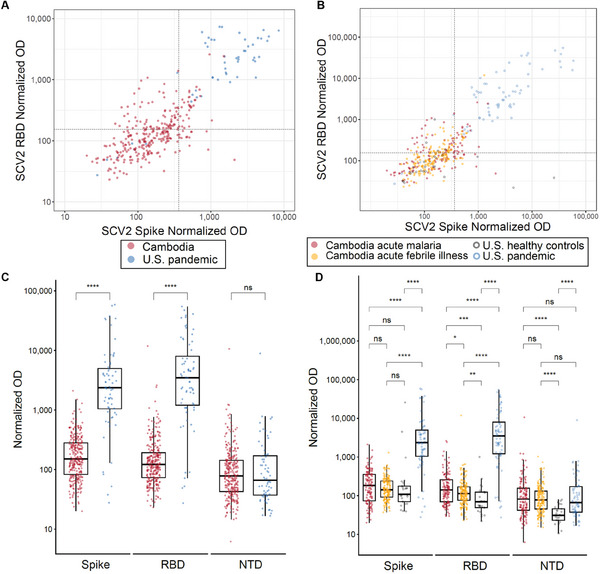
SCV2 seroreactivity in exposed U.S. persons and pre‐pandemic Cambodian persons. A) Total IgG (normalized OD) to SCV2 Spike and RBD in Cambodian pre‐pandemic (*N* = 293) and U.S. pandemic (*N* = 68) samples. The dotted lines indicate established assay cutoffs (3 standard deviations above the mean of archival negative controls). 32 Cambodian pre‐pandemic samples exhibit seropositivity for SCV2 based on Spike and RBD levels. B) Total IgG (normalized OD) to SCV2 Spike and RBD in Cambodian acute malaria cohort (*N* = 131), Cambodian acute undifferentiated febrile illness cohort (*N* = 162), and U.S. pandemic (*N* = 68) samples. The dotted lines indicate established assay cutoffs (3 standard deviations above the mean of archival negative controls). 22 samples from the Cambodian acute malaria cohort, 10 samples from the Cambodian undifferentiated febrile illness cohort, 61 samples from the U.S. pandemic cohort, and 0 samples from the U.S. pre‐pandemic cohort exhibit seropositivity to SCV2 based on Spike and RBD levels. C) Comparison of normalized OD for SCV2 Spike, RBD, and NTD antigens in Cambodian pre‐pandemic versus U.S. pandemic samples. Results of Wilcoxon rank sum test shown (*p*‐values: ^****^ <0.0001, ns ≥ 0.05). U.S. pandemic samples exhibit higher levels of reactivity to SCV2 Spike and RBD than Cambodian pre‐pandemic samples. D) Comparison of normalized OD for SCV2 Spike, RBD, and NTD antigens in Cambodian acute malaria cohort, Cambodian acute undifferentiated febrile illness cohort, and U.S. pandemic samples. Results of Wilcoxon rank sum test shown (*p*‐values: ^****^ < 0.0001, ns ≥ 0.05). There is no significant difference in reactivity to SCV2 Spike or NTD between the two Cambodian cohorts, although the acute undifferentiated Febrile illness cohort does have slightly lower SCV2 RBD reactivity than the acute malaria cohort.

In a prior study, we found SCV2 seroreactivity in 4–11% of malaria‐infected Cambodians sampled during 2005–2011.^[^ ^12^ ^]^ Here, we evaluated pre‐pandemic samples from 293 Cambodians, including a cohort with acute malaria (*N* = 131) and a second with acute undifferentiated febrile illness (*N* = 162). Using an established cutoff to whole Spike and RBD based on archival negative U.S. controls, 22/131 samples (16.8%) from the Cambodian acute malaria cohort and 10/162 (6.2%) samples from the Cambodian acute febrile illness cohort were seropositive for SCV2, accounting for a total seropositivity rate of 10.9%. In contrast, 61/68 (89.7%) U.S. pandemic samples and 0/25 U.S. healthy controls were seropositive for SCV2 (**Table** **1** and **Figure** **1**A,B). Antibody titers against SCV2 Spike and RBD, but not NTD, were higher in U.S. pandemic versus Cambodian samples while antibody titers against SCV2 RBD and NTD, but not whole Spike, were higher in Cambodian samples versus U.S. healthy controls (Figure 1C,D; Figures S3–S10, Supporting Information). A higher proportion of Cambodians in the acute malaria cohort had SCV2 antibody titers above the cutoff compared to the acute febrile illness cohort (p = 0.004). Importantly, the use of both Spike and RBD to determine seropositivity has been demonstrated to enable the identification of seropositive samples with high sensitivity and high specificity.^[^ ^21^ ^]^ As the data reported in this manuscript are optical density (OD) or normalized OD values, these values are not absolute measures of antibody concentration. Instances where RBD reactivity exceeds Spike reactivity may potentially be a result of differences in ELISA plate coating concentrations for these proteins.

**Table 1:** Seroreactivity of Cambodian pre‐pandemic and US pandemic samples to SCV2 antigens.

Threshold^a)^	Cambodian acute malaria cohort [*N* = 131]	Cambodian acute febrile illness cohort [*N* = 162]	*p*‐value^b)^	U.S. pandemic [*N* = 68]	U.S. healthy controls [*N* = 25]	*p*‐value^c)^
Spike only	32 (24%)	14 (9%)	<0.001	61 (90%)	3 (12%)	<0.001
RBD only	59 (45%)	51 (31%)	0.017	62 (91%)	4 (16%)	<0.001
Spike and RBD	22 (17%)	10 (6%)	0.004	61 (90%)	0	<0.001

^a)^ Threshold values are defined as three standard deviations above the mean of the archival negative controls^[^ ^21^ ^]^

^b)^ Pearson's Chi‐squared test comparing two Cambodian cohorts

^c)^ Pearson's Chi‐squared test comparing across all four cohorts (Cambodian and U.S.).

### 2.2 SCV2 Seroreactivity is not Entirely Explained by *Plasmodium spp*. Infection

**Figure 2:**
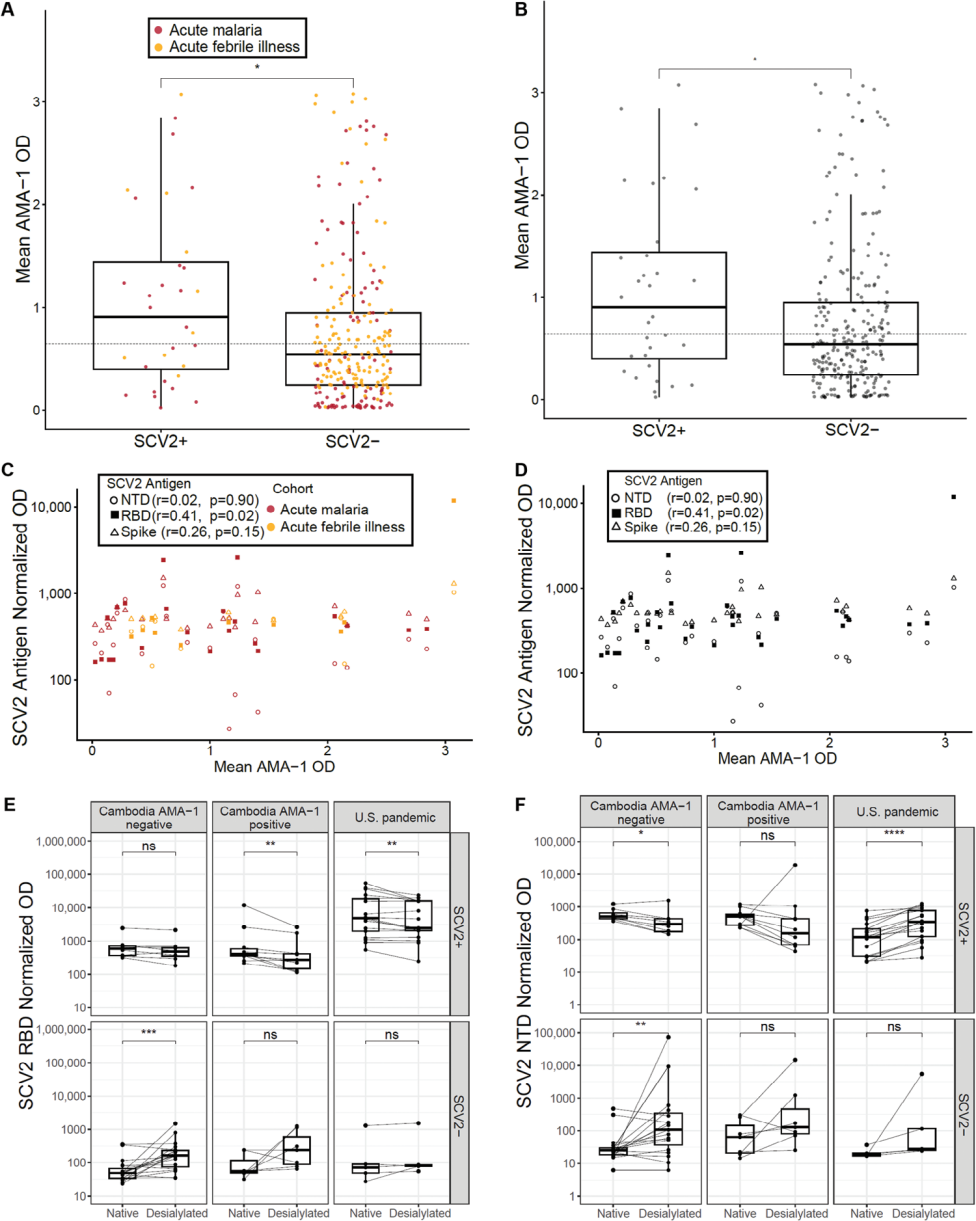
Malaria AMA‐1 positivity as a function of SCV2 seroreactivity A) AMA‐1 OD stratified by SCV2 seropositivity in 131 Cambodians living in a province with high malaria prevalence (acute malaria cohort) and 162 Cambodians living in a province with low malaria prevalence (acute febrile illness cohort). The dotted line indicates the cutoff for AMA‐1 positivity. B) AMA‐1 OD stratified by SCV2 seropositivity in both Cambodian cohorts combined. The dotted line indicates the cutoff for AMA‐1 positivity. SCV2‐positive samples exhibit slightly higher mean AMA‐1 reactivity than SCV2‐negative samples. C) SCV2 Spike, RBD, and NTD normalized OD by AMA‐1 OD in 32 Cambodian samples categorized by cohort (red = acute malaria cohort, yellow = acute febrile illness cohort). Spearman correlation coefficients and significance are shown in the inset. D) SCV2 Spike, RBD, and NTD normalized OD by AMA‐1 OD in 32 Cambodian samples not categorized by cohort. Spearman correlation coefficients and significance are shown in the inset. E) SCV2 RBD normalized OD of 66 samples before versus after neuraminidase treatment. Wilcoxon signed‐rank test *p*‐values shown. F) SCV2 NTD normalized OD of 66 samples before versus after neuraminidase treatment. Wilcoxon signed‐rank test *p*‐values shown (*p*‐values: ^*^ < 0.05, ^**^ <0.01, ^***^ <0.001, ^****^ <0.0001, ns ≥ 0.05).

We previously demonstrated a significant positive correlation between antibodies for SCV2 Spike and RBD and *Plasmodium falciparum* apical membrane antigen (AMA‐1).^[^ ^12^ ^]^ Here, we noted slightly higher SCV2 seropositivity among Cambodians in the acute malaria cohort versus the acute febrile illness cohort (**Figure** **2**A). As the latter cohort did not have accompanying diagnostics to isolate individuals with acute malaria, and neither cohort was standardized in sampling time relative to illness onset, which could impact the presence of potentially cross‐reactive antibodies, we elected to use antibodies against AMA‐1 as an indicator of an immune response raised against *P. falciparum*. We measured AMA‐1 antibodies in all 293 Cambodians and again found that AMA‐1 OD was higher in SCV2 positive versus negative samples (median OD 0.904 [IQR 0.400‐1.44] versus 0.544 [IQR 0.245‐0.950], p = 0.034). However, 43.8% (14/32) of SCV2 positive samples were AMA‐1 negative (Figure 2B). Within 32 SCV2 positive Cambodian samples, SCV2 RBD normalized OD weakly correlated with AMA‐1 OD (Spearman r 0.41, p = 0.02), and SCV2 Spike and NTD normalized OD did not correlate with AMA‐1 OD (Spearman r 0.26, p = 0.15 and r 0.023, p = 0.90, respectively) (Figure 2C,D).

To further explore whether SCV2 seropositivity could be a result of *Plasmodium* infection, we treated SCV2 protein constructs with neuraminidase to remove the sialic moieties that are the proposed targets of cross‐reactive antibodies (Figure S11, Supporting Information).^[^ ^19^ ^]^ We compared total IgG against SCV2 native and desialylated proteins in a 66‐sample subset of U.S. pandemic and Cambodian pre‐pandemic samples (Figures S12–S15, Supporting Information, selection detailed in Supporting Methods). Within 35 SCV2 positive samples (selected from the U.S. and Cambodian cohorts), antibody titers were numerically lower for desialylated antigens regardless of AMA‐1 positivity or country of origin, except for NTD normalized OD in U.S. samples which was higher for desialylated over native proteins (Figure 2C,D; Figure S16–S18, Supporting Information). These changes are potentially caused by changes in the accessibility of antigenic regions as a result of desilaylation, as glycosylation is known to play a role in protein conformation and the exposure of epitopes.^[^ ^22^ ^]^ Indeed, sialic acid residues are not unique to malaria and play important roles in other areas such as the HIV glycan shield.^[^ ^23^, ^24^, ^25^ ^]^ In the context of SCV2, sialic acids have been reported to have contradictory roles, with some reports indicating that sialic acids are involved in the binding of the ACE2 receptor with SCV2, while others have reported that they inhibit the binding of ACE2 with SCV2.^[^ ^26^, ^27^, ^28^, ^29^ ^]^ Fold‐changes in RBD normalized OD (desialylated:native) were similar across groups (AMA‐1‐positive Cambodian: median 0.639 [IQR 0.385‐0.728], AMA‐1‐negative Cambodian: median 0.859 [IQR 0.699‐0.925], U.S.: median 0.862 [IQR 0.464‐1.02]). Among this 66‐sample subset, 18 SCV2 seropositive Cambodian samples were identified; Of these 18 samples, RBD titers remained above the cutoff in 15 samples, even after neuraminidase treatment. Fold‐changes in NTD normalized OD (desialylated:native) were similar between AMA‐1 positive (median 0.356 [IQR 0.219‐0.617]) and AMA‐1 negative Cambodian samples (median 0.544 [IQR 0.402‐0.659]) but higher in U.S. samples (median 2.82 [IQR 1.87–3.64]). In 31 SCV2 negative samples, normalized OD against desialylated proteins was overall higher than that against native antigens, although within‐group differences were only significant for AMA‐1 negative Cambodians (Supplemental Data).

### 2.3 Cambodian Samples Exhibit Cross‐Reactivity to BatCoV and SCV2 Variant Antigens

**Figure 3:**
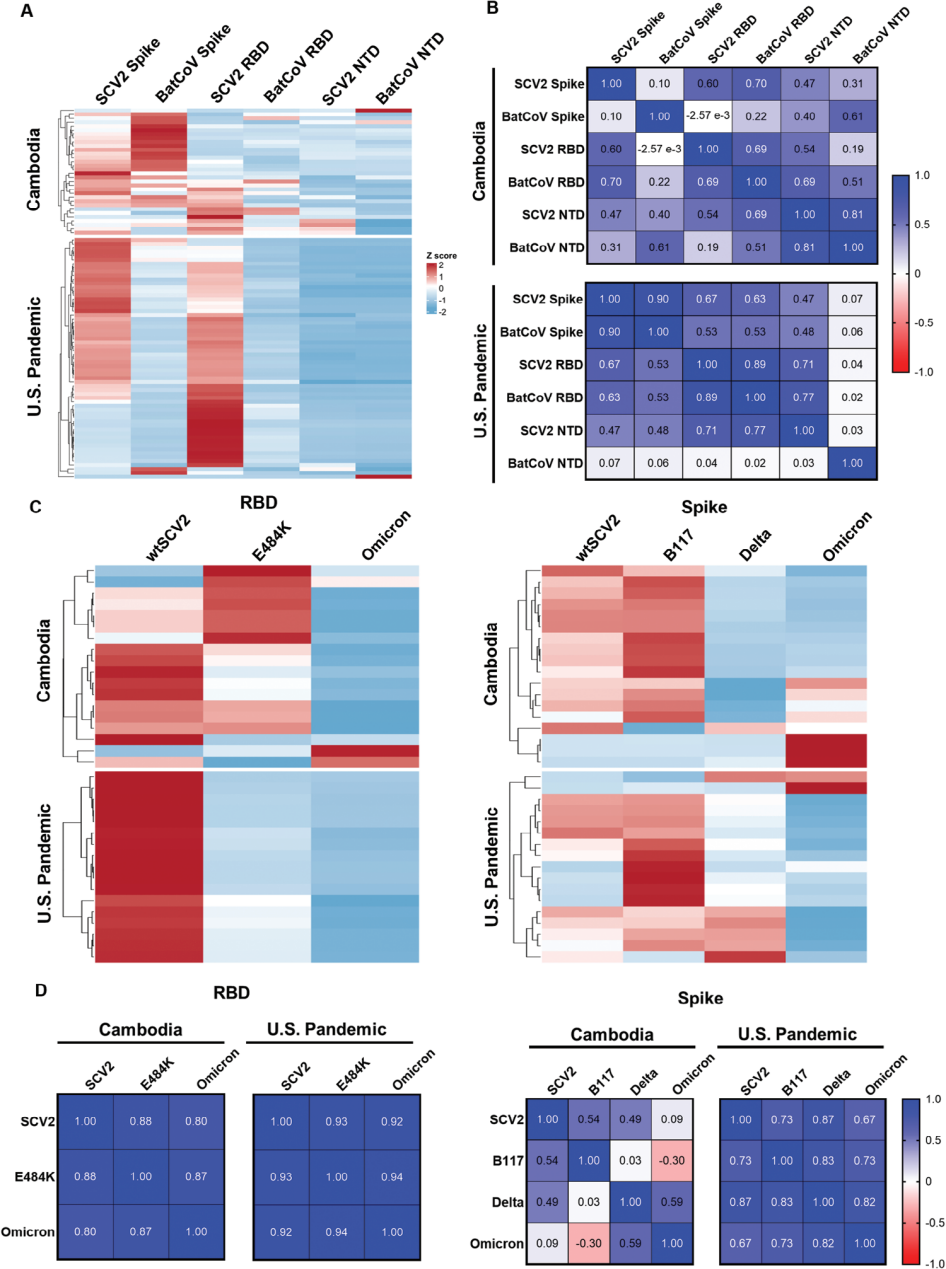
Seroreactivity of SCV2‐reactive samples to SCV2 and a Cambodian bat coronavirus. A) Heatmap of total IgG to SCV2 and BatCoV protein constructs in SCV2‐reactive Cambodian samples (top, *N* = 32) and U.S. samples (bottom, *N* = 61). BatCoV Spike had the highest antibody titers in over half of the Cambodian samples, while most U.S. samples had the highest titers to SCV2 RBD. B) Correlation matrices for total IgG to SCV2 and BatCoV protein constructs in SCV2‐reactive Cambodian samples (top) and U.S. samples (bottom). A strong positive correlation was observed between BatCoV NTD and SCV2 NTD for seropositive Cambodian samples. A strong positive correlation was observed between SCV2 SPIKE and BatCoV SPIKE, as well as between SCV2 RBD and BatCoV RBD for seropositive U.S. pandemic samples. C) Heatmaps of total IgG to different SCV2 RBD (left) and Spike (right) constructs for wild‐type (wt) SCV2 and variants B.1.1.7, B.1.617.2, B.1.1.529, and E484K. U.S. samples had higher reactivity to ancestral SCV2 RBD than to E484K or B.1.1.529 variants, while Cambodian samples had varied reactivity to RBD and Spike proteins across the different variants. D) Correlation matrices for total IgG to different SCV2 RBD (left) and Spike (right) constructs for wild‐type (wt) SCV2 and variants B.1.1.7, B.1.617.2, B.1.1.529, and E484K. A high degree of correlation is observed for reactivity to RBD in wild‐type SCV2 and variants E484K and B.1.1.529. For Spike, higher levels of correlation between reactivity to wild‐type SCV2 and variants were observed in U.S. Pandemic samples than in Cambodian samples.

Since a large portion of the Cambodian pre‐pandemic cross‐reactivity cannot be explained by *Plasmodium* infection, we next sought to determine whether prior exposure to SCV2‐related bCoVs may be contributing to SCV2 cross‐reactivity. We synthesized whole Spike, RBD, and NTD proteins from BatCoV, a sarbecovirus isolated from a bat collected in Cambodia in 2010, and tested the reactivity of all 32 SCV2 seropositive Cambodian samples and 61 SCV2 seropositive US samples with BatCoV antigens (Figures S6–S8 and S10, Supporting Information). Given the small number of SCV2 seropositive Cambodian samples, and the lack of clinical and demographic data by which to clearly distinguish subgroups, both Cambodian cohorts were analyzed together. Among 6 SCV2 and BatCoV protein constructs, BatCoV Spike elicited the highest antibody titers in 17/32 (53.1%) Cambodian samples, followed by SCV2 RBD in 7/32 (21.9%) and SCV2 SPIKE in 5/32 (15.6%). In contrast, most U.S. samples had the highest titers to SCV2 RBD (34/61; 55.7%) and SCV2 Spike (24/61; 39.3%) (**Figure** **3**A). For seropositive Cambodian samples, a strong positive correlation was observed between BatCoV NTD and SCV2 NTD. For seropositive U.S. samples, strong positive correlations were observed between SCV2 SPIKE and BatCoV SPIKE, as well as between SCV2 RBD and BatCoV RBD (Figure 3B). To explore whether antibodies bound preferentially to BatCoV over SCV2, we pre‐incubated Cambodian sera with BatCoV Spike before performing a regular ELISA using SCV2 Spike. A decrease in SCV2 Spike normalized OD was seen in 28/32 (87.5%) samples. The median fold‐change between normalized OD values for samples pre‐incubated with BatCoV Spike versus those not pre‐incubated was 0.661 (IQR 0.437 – 0.854), suggesting that some SCV2 Spike cross‐reactive antibodies within Cambodian sera bind to BatCoV Spike.

Based on these findings, we hypothesized that some of the SCV2 cross‐reactivity in Cambodian sera may have resulted from prior exposure to different SCV2‐related bCoVs. We explored antibody titers to different SCV2 variants in a subset of Cambodian and U.S. samples (Figures S19–S23, Supporting Information). U.S. samples uniformly had the highest titers to ancestral SCV2 RBD (over E484K and B.1.1.529 variants) but varied responses to different whole Spike constructs (highest reactivity to B.1.1.7 in 10/17 [58.8%]), likely explained by the dominant immunogenicity of RBD^[^ ^30^ ^]^ paired with high genetic and antigenic variability of this region among SCV2 variants. In contrast, Cambodian samples had varied responses to both RBD and Spike constructs, suggesting a range of antigenically distinct exposures (Figure 3C). For both Cambodian and U.S. samples, highly positive correlations are observed between reactivity against SCV2 RBD, E484K, and B.1.1.529 (Figure 3D). A highly positive correlation is also observed for reactivity to SCV2 SPIKE and B.1.1.7, SCV2 SPIKE and Delta, B.1.1.7 and Delta, Omicron and Delta, and B.1.1.7 and Omicron for U.S. samples. In contrast, the same highly positive correlation between different SPIKE constructs is not observed for Cambodian samples.

### 2.4 Cambodian Samples Exhibit Some Neutralizing Activity Against SCV2 and bCOV Antigens

**Figure 4:**
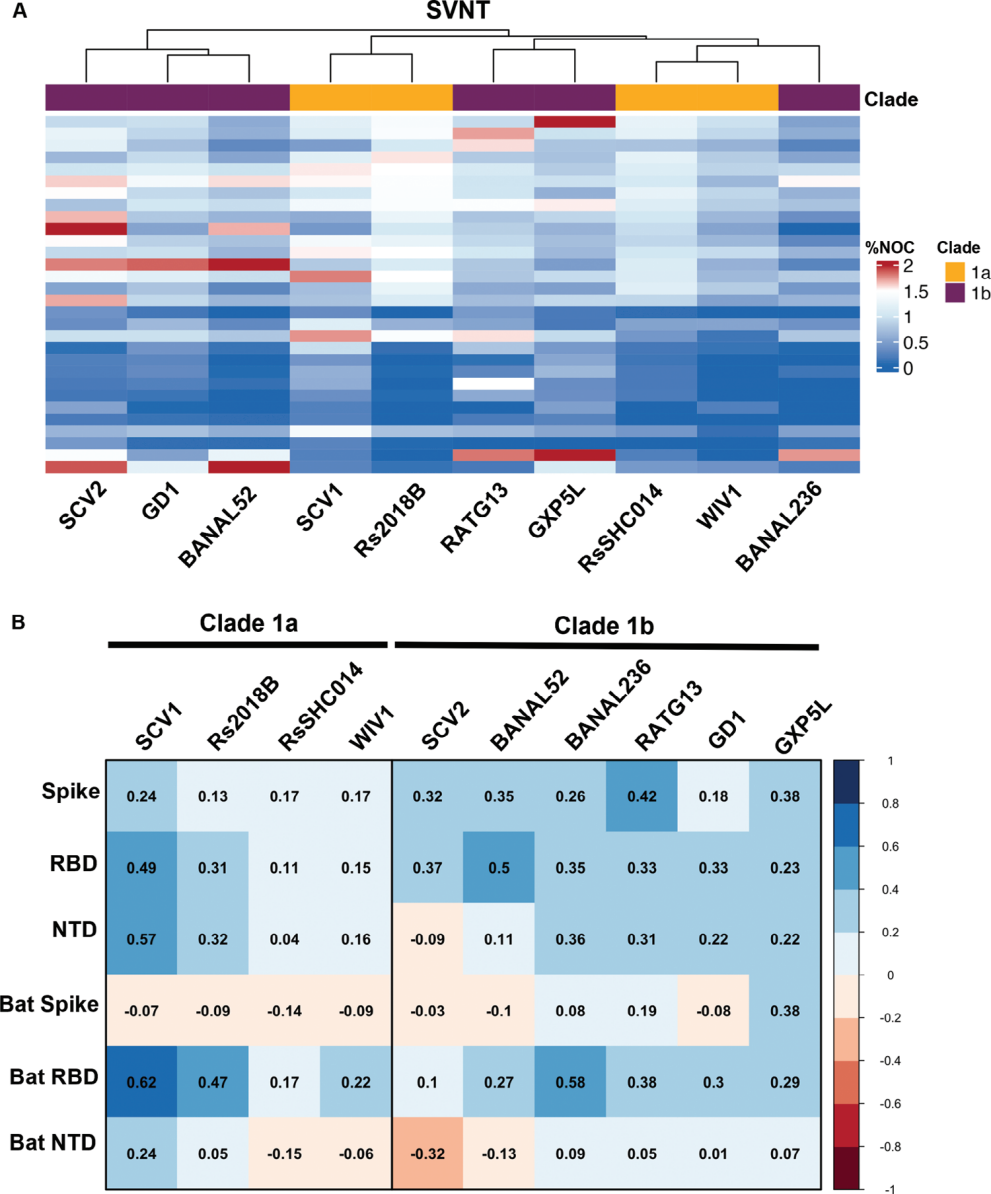
Surrogate virus neutralization in Cambodian serum. A) Heatmaps of sVNT titers of 30 SCV2 ELISA‐positive Cambodian samples. Cell values represent fold‐change of percent inhibition over dynamic cutoff; positive threshold set at 1.5 (red indicates values >1.5, blue indicates values <1.5). 8 SCV2‐seropositive Cambodian samples had neutralizing activity for SCV2. 14 SCV2‐seropositive Cambodian samples had neutralizing activity against at least one clade 1a or 1b bCoV. B) Correlation matrix of sVNT (columns) and ELISA (rows) titers in 43 Cambodian samples with positive correlations denoted in blue and negative correlations in red. Numbers indicate Spearman correlation coefficients. Seroreactivity to SCV2 RBD IgG was significantly correlated with SCV1 and BANAL52 neutralization, suggesting that the presence of neutralizing antibodies against similar viruses can lead to SCV2 reactivity.

Next, we explored the breadth of bCoV and SCV2‐specific immunity in our Cambodian cohort. We selected 30 SCV2 positive and 13 SCV2 negative pre‐pandemic Cambodian samples and performed surrogate viral neutralization tests (sVNT) against a mixed panel of clade 1a (SCV1, Rs2018B, RsSHC014, WIV‐1) and 1b (SCV2, BANAL52, BANAL236, RaTG13, GD.1, GX.P5L) bCoVs. Using a threshold of 1.5 times the percent neutralization over dynamic assay cut‐off values, we detected SCV2 neutralizing activity in 8/30 (26.7%) SCV2 seropositive Cambodian samples (**Figure** **4**A). Two of 13 SCV2 seronegative Cambodian samples had SCV2 neutralizing activity detected; both these samples had SCV2 RBD ELISA values above assay cutoff but SCV2 Spike ELISA values below the cutoff (Table S2, Supporting Information). SCV2 neutralizing activity correlated modestly with total IgG titers for SCV2 RBD (Spearman r 0.37, p = 0.04) and Spike (Spearman r 0.32, p = 0.09) but poorly with NTD (Spearman r −0.09, p = 0.63). Neutralization of any clade 1a or 1b bCoV was seen in 14/30 (46.7%) SCV2 seropositive samples (**Table** **2**), most commonly SCV1 (*N* = 5), followed by bat bCoVs BANAL52 (*N* = 4) and RaTG13 (*N* = 4), and pangolin bCoV GX.P5L (*N* = 3). BatCoV RBD IgG titers were significantly correlated with SCV1 (Spearman r 0.62, p < 0.01), BANAL236 (Spearman r 0.58, p < 0.01), Rs2018B (Spearman r 0.47, p = 0.01) and RaTG13 (Spearman r 0.38, p = 0.04) neutralizing activity, while SCV2 RBD IgG titers were significantly correlated with SCV1 (Spearman r 0.49, p = 0.01) and BANAL52 (Spearman r 0.50, p = 0.01) neutralization (Figure 4B).

**Table 2:** Surrogate virus neutralization test positivity in SCV2 ELISA positive and negative pre‐pandemic Cambodian samples.

bCoV sVNT			SCV2 ELISA	
Clade	Virus	No. positive [%, 95% Confidence Interval^a)^]	Positive (*N* = 30) [%, 95% Confidence Interval^a)^]	Negative (*N* = 13) [%, 95% Confidence Interval^a)^]
1b	SCV2	10 [23.3%, 13.2–37.7%]	8 [27%, 14.2–44.4%]	2 [15.4%, 2.7–42.2%]
1b	BANAL52	4 [9.3%, 3.7–21.6%]	4 [13.3%, 5.3–29.7%]	0 [0%, 0–22.8%]
1b	BANAL236	3 [7%, 2.4–18.6%]	2 [6.7%, 1.2–21.3%]	1 [7.7%, 0.4–33.3%]
1b	GD.1	1 [2.3%, 0.1–12.1%]	1 [3.3%, 0.2–6.7%]	0 [0%, 0–22.8%]
1b	GX.P5L	4 [9.3%, 3.7–21.6%]	3 [10%, 3.5–25.6%]	1 [7.7%, 0.4–33.3%]
1b	RaTG13	4 [9.3%, 3.7–21.6%]	4 [13.3%, 5.3–29.7%]	0 [0%, 0–22.8%]
1a	Rs2018B	1 [2.3%, 0.1–12.1%]	1 [3.3%, 0.2–6.7%]	0 [0%, 0–22.8%]
1a	RsSHC014	0 [0%, 0–8.2%]	0 [0%, 0–11.4%]	0 [0%, 0–22.8%]
1a	SCV1	7 [16.3%, 8.1–30%]	5 [16.7%, 7.3–33.6%]	2 [15.4%, 2.7–42.2%]
1a	WIV1	0 [0%, 0–8.2%]	0 [0%, 0–11.4%]	0 [0%, 0–22.8%]
1b	Any 1b bCoV	17 [39.5%, 26.4–54.4%]	14 [46.7%, 30.2–53.9%]	3 [23.1%, 8.2–50.3%]
1a	Any 1a bCOV	8 [18.6%, 9.7–32.6%]	6 [20%, 9.5–37.3%]	2 [15.4%, 2.7–42.2%]
Both	Any bCoV	22 [51.2%, 36.8–65.4%]	18 [60%, 42.3–75.4%]	4 [30.8%, 12.7–57.6%]

^a)^ Wilson/Brown 95% Confidence Intervals.

## 3 Discussion

This study demonstrates pre‐pandemic humoral reactivity to SCV2 and to a representative Cambodian horseshoe bat BatCoV in Cambodian people, a finding not entirely explained by prior infection with *falciparum* malaria. Neutralizing activity against various human and animal betacoronaviruses suggests that functional antibodies may have arisen from prior immune challenges with related bCoV, including from zoonotic reservoirs. These data are striking in the context of the lack of SCV2‐related morbidity and mortality notable in Cambodia and the Greater Mekong Subregion with ancestral virus in 2020,^[^ ^13^, ^31^ ^]^ and the continued uncertainty regarding the origins of SCV2.^[^ ^32^ ^]^

Prior reports have found a high seroprevalence of antibodies reacting with SCV2 in regions with exposure to malaria, dengue, and other diseases.^[^ ^12^, ^19^, ^33^, ^34^, ^35^ ^]^ Among potential mechanisms, in silico studies have identified shared immunodominant epitopes with cross‐immunogenic reactivity between *P. falciparum* and SCV2 antigens.^[^ ^36^ ^]^ One study examined cross‐reactive antibodies to the SCV2 Spike protein S1 domain in a cohort with acute *falciparum* malaria,^[^ ^19^ ^]^ although cross‐reactivity did not appear to extend to SCV2 RBD. Treating the S1 protein with neuraminidase appeared to completely abolish antibody responses, suggesting that cross‐reactivity was due to antibodies binding with the terminal sialic acids of complex glycans in SCV2 Spike S1.^[^ ^19^ ^]^ In contrast, we found SCV2‐reactive antibodies for both SCV2 whole Spike and RBD constructs in Cambodian participants with and without acute or prior malaria exposure. Neuraminidase treatment only minimally reduced serum reactivity and reductions in reactivity were similar regardless of AMA‐1 reactivity (a measure of the *P. falciparum*‐targeted immune response). Similarly, serosurveys in two rural communities in Tanzania found that SCV2 Spike IgG during the pandemic was not associated with coinciding malaria infections or previous parasite exposure.^[^ ^37^ ^]^ These data suggest that while some cross‐reactivity between *P. falciparum* and SCV2 may exist, this may not fully explain pre‐pandemic SCV2 reactivity in Cambodians. Further, any cross‐reactive antibodies appear to lack functionality^[^ ^12^ ^]^ potentially due to loose binding,^[^ ^34^ ^]^ making this an unlikely mechanism for bCoV neutralization and immunity.

Another possible explanation for the high reactivity of pre‐pandemic Cambodian samples against SCV2 proteins could be prior exposure to zoonotic SCV2‐related bCoV. The genus *Rhinolophus* are natural reservoir of bCoV.^[^ ^38^ ^]^ The hunt for the origins of SCV2 has led to the discovery of a number of bat bCoV with varying levels of homology to SCV2. Early reports of SCV2 noted high sequence identity (96.2%) with RaTG13, a bCoV isolated from *Rhinolophus affinis* in Yunnan Province, China in 2013.^[^ ^39^ ^]^ Other SCV2‐related bCoVs have since been described in different *Rhinolophus* species in Southeast Asia, including two in Steung Treung province in Cambodia (RshSTT182 and RshSTT200), which share 92.6% sequence homology with SCV2,^[^ ^20^ ^]^ and another in Chachoengsao province of Thailand (RacCS203), with 95.9% sequence homology with SCV2.^[^ ^40^ ^]^ However, receptor binding studies failed to demonstrate successful binding of RshSTT200 or RacCS203 RBD to the human ACE2 receptor. Viruses that lack affinity for the human ACE2 receptor (hACE) and are unlikely candidates to jump the species barrier and infect humans; spillover would have required recombination events and/or passage through an intermediate host to acquire the correct binding proteins to mediate entry into human cells.^[^ ^41^ ^]^ More recently, a study sampling bats in northern Laos found 3 bCoVs (BANAL‐52, BANAL‐103, and BANAL‐236) in *Rhinolophus* individuals with high similarity to human SCV2.^[^ ^42^ ^]^ BANAL‐52 has demonstrated the highest nucleotide identity (96.8%) of any bat bCoV with SCV2 to date. BANAL‐236 Spike‐pseudotyped lentivirus demonstrated Spike‐mediated entry into hACE2‐expressing cells that could be blocked by human sera neutralizing SCV2 but not control sera. While evidence remains circumstantial, these data drive the hypothesis that bat‐to‐human spillover may have led to the emergence of SCV2.

Here, we challenged Cambodian and U.S. sera that were seroreactive for SCV2 with protein constructs representing Spike, RBD, and NTD from RshSTT182, which we used as a representative virus. Although there have been no confirmed human infections with RshSTT182 and its Spike protein is not compatible with the human ACE2 receptor, its high sequence homology to SCV2 and its discovery in a Rhinolophus individual from Cambodia made it an attractive option as a representative virus in our study of Cambodian sera. We found that Cambodian sera was equally or more reactive to BatCoV antigens than SCV2 antigens, in contrast with U.S. pandemic sera which had higher responses to SCV2 than BatCoV antigens. These findings do not necessarily mean that the Cambodian cohort was exposed to RshSTT182, but rather that they may have had prior exposure to similar viruses with sequence homology to SCV2.

Aside from Cambodian samples exhibiting reactivity to SCV2 antigens, we also found strong positive correlations between their reactivity to SCV2 Spike and BatCoV RBD, and SCV2 NTD and BatCoV NTD. These findings further support our hypothesis of cross‐reactivity between anti‐SCV2 antibodies and antibodies that may have formed against other similar coronaviruses. This is further supported by the strong positive correlation between reactivity to SCV2 Spike and BatCoV Spike, as well as between SCV2 RBD and BatCoV RBD, in seropositive U.S. pandemic samples.

The Spike protein consisting of S1 RBD, S1 NTD, and S2 is the most highly variable region of the SARS‐related bCoVs. Within Spike, NTD is the most variable region,^[^ ^42^ ^]^ and RBD is the most immunogenic.^[^ ^30^, ^43^ ^]^ Correspondingly, we noted higher RBD than NTD responses to both SCV2 and BatCoV in U.S. individuals sampled during the pandemic, although curiously the ratio of BatCoV NTD to SCV2 NTD responses was similar in SCV2‐reactive U.S. and Cambodian samples. While we hypothesize that SCV2 seroreactivity in some Cambodians may be explained by exposure to related bCoVs, these past exposures may share homology in RBD but diverge at NTD from RshSTT182. Our cohort of SCV2 seroreactive Cambodians demonstrated stronger responses to B.1.1.7 but lower responses to B.1.617.2 and B.1.1.529 compared to wild‐type SCV2. This may suggest that past bCoV exposure more closely resembled B.1.1.7 than the original SCV2 strain in Wuhan; such pre‐pandemic immunity could have explained the apparent protection against penetration of the Alpha variant but not later Delta and Omicron variants in Cambodia and the greater Mekong.^[^ ^31^ ^]^ Specifically, Cambodia reported fewer than 10 monthly cases per million persons through January 2021; case numbers burgeoned in the following months with the arrival of the Delta wave and peaked at 1500 per million persons in July 2021 despite the continuation of stringent pandemic shutdown measures.^[^ ^13^, ^44^, ^45^ ^]^ In contrast, U.S. sera from individuals living in New York City had almost universally the strongest responses to wild‐type SCV2 RBD, consistent with this representing the dominant exposure in the summer of 2020.

The limitations of the study include convenience sampling of Cambodian and U.S. cohorts, as is often necessary for retrospective pre‐ and early pandemic studies. The lack of metadata on the exact timing of U.S. individuals’ SCV2 infections does not allow for a finer understanding of the evolution or decay of their antibody response. Similarly, we do not have detailed zoonotic risk exposure profiles for the Cambodian individuals, and extrapolate from the overarching assumption that zoonotic exposure to bats is higher in Cambodia than in the metropolitan U.S. We also do not have acute febrile surveillance data demonstrating infection of sampled Cambodians with zoonotic bCOVs. In this work using two adventitiously sampled cohorts we did not seek to identify the specific zoonotic bCOVs to which the Cambodian population may have been exposed, some of which are likely undiscovered, but rather to evaluate potential antibody cross‐reactivity between these unknown viruses and SCV2. In the absence of knowledge of exact virus exposures, we have challenged Cambodian sera with a variety of antigens from known bCoVs; in doing so, we hoped to detect past infections with these viruses or others closely related to them and eliciting cross‐reactive immunity. Of note, the antigens used in ELISA and SVNT experiments were from different bCoVs, but this should not detract from the overall message that this population is exposed to a wide breadth of zoonotic viruses, the combination of which may have contributed to pre‐pandemic SCV2 seropositivity. Further studies would be needed to support novel bCoV discovery and determine the mechanisms of the cross‐reactivity observed here, which may include aspects related to epitope and antigenic distance and are out of the scope of this work. Further experiments in which the competitive ELISA is repeated for U.S. pandemic sera pre‐incubated with BatCoV or Cambodian sera are pre‐incubated with SCV2 Spike and then an ELISA for BatCoV Spike is conducted, would provide further support for these results, but are out of the scope of this work.

We expect this discovery to provide a platform from which to better understand and explore the origins of humoral immunity to SCV2 and closely related betacoronaviruses in individuals from diverse pathogen exposure landscapes. Enhanced surveillance of bat guano farms combined with population‐scale serosurveillance of rural Mekong populations will be essential countermeasures to prevent the emergence, or re‐emergence, of these viruses in the future. These findings also indicate that prior pathogen exposure should be considered in the assessment of antibody levels against novel viruses, and the role of cross‐reactive antibodies, as well as their potential neutralizing properties, should be evaluated. Incorporation of this information into pandemic modeling approaches in the future can serve to more accurately predict the spread of viruses among different populations based on their diverse viral exposure profiles.

## 4 Materials Section

### Study Samples

This study used biobanked serum samples from individuals living in Cambodia (total *N* = 293) and the U.S. (*N* = 68). Data on patient demographics and clinical outcomes were not provided for these cohorts. The first Cambodian cohort (*N* = 131) included individuals in Pursat province acutely infected with *Plasmodium falciparum* that were enrolled in a malaria research study between 2005 and 2011 (NCT00341003). The second Cambodian cohort (*N* = 162) included individuals presenting with acute undifferentiated febrile illness to a tertiary referral hospital in Kampong Speu province in 2019 and early 2020, before detected community transmission of SCV2 in Cambodia (NCT04034264). U.S. samples were collected during a period of high community transmission of SCV2 (summer 2020) from symptomatic individuals in New York City as part of a local clinic blood drive. Negative sera controls (*N* = 25) were derived from protocols 18‐I‐0101 and 99‐CC‐0168. Use of de‐identified samples for this study was approved by the institutional review board of the National Institute of Allergy and Infectious Diseases and the National Ethics Committee on Human Research in Cambodia.

### Virus Protein Preparation

Soluble recombinant Spike proteins were created of SCV2 and bat coronavirus RshSTT182 (BatCoV). For both SCV2 and BatCoV, ancestral virus S1 (“Spike”) proteins (individual constructs for whole Spike, receptor binding domain [RBD], and N‐terminal domain [NTD]) were created as previously described.^[^ ^46^ ^]^ In addition, five constructs representing SCV2 variants/mutants (whole Spike of B.1.1.7, B.1.617.2, and B.1.1.529, and RBD of B.1.1.529 and E484K), and neuraminidase‐treated desialylated protein constructs of ancestral SCV2 RBD and NTD, were created and tested in a subset of samples (Supplement).

### Betacoronavirus Enzyme‐Linked Immunosorbent Assays

To screen for bCoV immunity, IgG against SCV2 and BatCoV antigens was detected using an enzyme‐linked immunosorbent assay (ELISA). Experiments were conducted in an automated fashion, but the analysis was unblinded. Briefly, 384‐well plates were coated with synthesized bCoV proteins overnight at 4 °C (50 µL per well), then washed three times with 100 µL PBST (0.5% Tween in 1X PBS) using a BioTek EL 406 washer dispenser, blocked with 100 µL blocking buffer (5% non‐fat dairy milk in PBST) for 2 h, then washed three times with PBST. Serum samples were initially diluted at 1:400 in blocking buffer, then serially to obtain dilutions of 1:800, 1:1600, 1:3200, 1:6400, 1:12800, 1:25600, and 1:51200. Samples were added in duplicate (50 µL each) to prepared plates. Anti‐SCV2 S1 RBD monoclonal antibody (GenScript) and buffer‐only solution were used as positive and negative controls, respectively. Plates were incubated for 1 h at room temperature, washed with PBST three times, followed by the addition of 50 µL IgG secondary antibody solution (horseradish peroxidase‐conjugated goat anti‐Human IgG, ThermoFisher Scientific) and incubation at room temperature for 1 h. Each well was washed three times with PBST, following which 30 µL of substrate (1‐Step Ultra TMB‐ELISA Substrate Solution, ThermoFisher Scientific) was added. After incubation for 10 min, 30 µL of stop solution (ELISA Stop Solution, Invitrogen) was added to each well, and plates were immediately read on a BioTek Epoch 2 microplate reader. Absorbance was read at 450 nm (A450) and 650 nm (A650) and the final optical density (OD) value for each well was determined by subtracting A650 from A450 to remove the background signal. Normalized OD values for samples were calculated by multiplying OD by the dilution factor, averaged over duplicates. If the OD of both duplicates fell outside the assay's linear range (range 1 to 3), the value closest to the linear range was taken and multiplied by its dilution factor. A total of three samples were excluded from the analysis (one duplicated Cambodian sample and two control samples). For the excluded control samples, one had erroneously been identified as a healthy control and the other had high cross‐reactivity for seasonal coronaviruses. Previously, we've found cross‐reactivity between seasonal betacoronaviruses and SCV2.^[^ ^47^ ^]^

### Competitive Enzyme‐Linked Immunosorbent Assays

A competitive ELISA was performed in SCV2‐seropositive Cambodian samples to compare the relative affinity of detected IgG for SCV2 versus BatCoV Spike antigens. Briefly, 96‐well plates were coated with synthesized SCV2 Spike protein overnight at 4 °C (50 µL per well at a concentration of 1 µg mL^−1^), then washed three times with 100 µL PBST (0.5% Tween in 1X PBS) using a Molecular Devices Skanwasher 400 dispenser, blocked with 200 µL blocking buffer (5% non‐fat dairy milk in PBST) for 2 h, then washed three times with PBST. Serum samples were initially diluted at 1:400 in blocking buffer, and incubated for 30 min at RT with 5 µg mL^−1^ of RshSTT182 Spike or BSA (Sigma). Samples were added in duplicate (50 µL each) to prepared plates. Plates were incubated for 1 h at room temperature, washed with PBST three times, followed by the addition of 50 µL IgG secondary antibody solution (Alkaline phosphatase‐conjugated goat anti‐Human IgG, Sigma) and incubation at room temperature for 1 h. Each well was washed three times with PBST, following which 50 µL of the substrate (pNPP Substrate Solution, Sigma) was added. After incubation for 30 min plates were immediately read on a Molecular Devices microplate reader. Absorbance was read at 405 nm (A405). Normalized OD values for samples were calculated by multiplying OD by the dilution factor, averaged over duplicates. If the OD of both duplicates fell outside the assay's linear range (range 1 to 3), the value closest to the linear range was taken and multiplied by its dilution factor.

### *Plasmodium spp* Enzyme‐Linked Immunosorbent Assay

Prior exposure to *Plasmodium* was determined via detection of IgG against AMA‐1, a membrane protein expressed in the merozoite stage that facilitates parasite invasion into red blood cells. AMA‐1 protein production and ELISA were performed as previously described^[^ ^48^ ^]^ with a few modifications: samples were diluted at 1:500 and loaded in triplicate (100 µL each) onto prepared plates. For the secondary antibody solution, horseradish peroxidase‐conjugated anti‐Human IgG secondary antibody (ThermoFisher Scientific) was used at 1:45000 dilution. Incubation with substrate solution (1‐Step Ultra TMB‐ELISA Substrate Solution, ThermoFisher Scientific) was performed for 10 min. The reaction was stopped with an equal volume of stop solution (ELISA Stop Solution, Invitrogen) and absorbance read at 450 nm using a SpectraMax M3 plate reader.

### Surrogate Virus Neutralization Tests

To detect i) the breadth of bCoV‐specific immune signatures and ii) the functionality of reactive and/or cross‐reactive antibodies, surrogate virus neutralization tests (SVNT) were performed against an established panel of 2 human bCoVs (SCV2 and SCV1) and 8 animal bCoVs (WIV1, RsSHC014, Rs2018B, BANAL52, BANAL236, RaTG13, GX‐P5L, and GD‐1).^[^ ^14^, ^49^ ^]^ A subset of 43 Cambodian samples from this study and a prior serosurvey^[^ ^12^ ^]^ was selected (Supplement) for shipment to the National University of Singapore where SVNT was performed. Briefly, AviTag‐biotinylated RBD proteins from 10 bCoVs were coated on MagPlex‐Avidin microspheres (Luminex) and preincubated with sera prior to the addition of phycoerythrin‐conjugated hACE2, to detect antibody‐mediated blockage of the interaction between hACE2 and viral RBD.

### Statistical Analysis

Spearman correlation matrices of SCV‐2 positive samples and Wilson/Brown confidence interval calculations for surrogate virus neutralization assays were conducted in GraphPad Prism 10. Optical density normalization was conducted in Matlab. All other analyses were conducted using R version 4.3.1.^[^ ^50^ ^]^ An overview of sample selection and sample sizes used for each analysis is provided in Figures S1 and S2 (Supporting Information). No a priori calculations were conducted to determine sample size. However, sample sizes were determined based on previous work that established the number of negative controls needed as part of the development of the SCV2 ELISA assay.^[^ ^21^ ^]^

## Conflict of Interest

The authors declare no conflict of interest.

## Supporting information



Supporting Information

## Data Availability

The data that support the findings of this study are available from the corresponding author upon reasonable request.
