# Supplementary material for: Humoral Immunity Profiling to Pandemic and Bat‐Derived Coronavirus Variants: A Geographical Comparison
Source: Adv Sci (Weinh). 2024 Oct 29;12(1):2403503. doi: 10.1002/advs.202403503 (PMC11714182; doi:10.1002/advs.202403503)
Supplement: Supplementary file 1 — Supporting Information [file ADVS-12-2403503-s001.docx]

Supporting Information

Humoral immunity profiling to pandemic and bat-derived coronavirus variants: a geographical comparison

Parinaz Fathi^§1^, Andrea Lucia Alfonso^§1^, Christina Yek^§2^, Zoe Putman^3^, Matthew Drew^3^, Dominic Esposito^3^, Irfan Zaidi^4^, Sophana Chea^5^, Sokna Ly^5^, Rathanak Sath^5^, Chanthap Lon^5^, Huch Chea^6^, Rithea Leang^6^, Rekol Huy^6^, Sovann Ly^7^, Heng Seng^7^, Chee Wah Tan^8,9^, Feng Zhu^8^, Lin-Fa Wang^8^, Fabiano Oliveira^2^, Kaitlyn Sadtler^1*^, Jessica Manning^2,5,10*^

**Supplemental Methods**

**Table S1.** Summary of 16 antigens used in betacoronavirus ELISA assays

| **Antigen** | **Plate Coating Concentration [µg/mL]** | **Samples Tested** |
| --- | --- | --- |
| SCV2 Spike | 1 | All samples |
| SCV2 RBD | 2 | All samples |
| SCV2 NTD | 2 | All samples |
| BatCoV Spike | 1 | All samples |
| BatCoV RBD | 2 | All samples |
| BatCoV NTD | 2 | All samples |
| E484K (RBD) | 2 | All samples |
| B.1.1.7 Spike | 2 | All samples |
| Nucleocapsid | 2 | All Samples |
| Desialylated SCV2 RBD | 2 | 66 select samples |
| Desialylated SCV2 NTD | 2 | 66 select samples |
| Desialylated BatCoV RBD | 2 | 66 select samples |
| Desialylated BatCoV NTD | 2 | 66 select samples |
| B.1.617.2 Spike | 1 | 66 select samples |
| B.1.1.529 Spike | 1 | 66 select samples |
| B.1.1.529 RBD | 2 | 66 select samples |

*Selection of 66 samples for SCV2 variant and desialylated protein ELISA*

The 66-sample subset consisted of 22 samples from the Cambodian cohort with acute malaria, 22 samples from the Cambodian cohort with undifferentiated acute fever, and 22 samples from the U.S. pandemic cohort. Each set of 22 was composed of 11 high-OD and 11 low-OD samples. To identify these specific samples, the following guidelines were used. For each of the three cohorts, the 25 samples with highest OD values (evaluated at the 1:400 sample dilution) each for SCV2 RBD, SCV2 NTD, BatCoV RBD, and BatCoV NTD were chosen. The samples that overlapped between the high SCV2 RBD and high SCV2 NTD lists were identified, as well as those that overlapped between the high BatCoV RBD and high BatCoV NTD lists. From these lists, the samples that overlapped between the SCV2 antigens and the BatCoV antigens were identified. In cases where there were already 11 samples that overlapped between all the RBDs and NTDs of interest, those 11 samples were chosen for the subset. In cases where there were less than 11 samples that overlapped between all the RBDs and NTDs of interest, those samples were selected and then others from the list of samples that overlapped between high BatCoV RBD and high BatCoV NTD lists were chosen manually. To identify the 11 low-OD samples for each sample category, the above process was repeated with the 25 samples with lowest OD each for SCV2 RBD, SCV2 NTD, BatCoV RBD, and BatCoV NTD.

*Selection of 43 samples for surrogate viral neutralization tests*

The 43-sample subset consisted of a random selection of 18 samples from the current Cambodian cohort and 25 samples from a prior cohort of Cambodians with acute malaria (Manning, EID 2022). Of these, 30 were SCV2 seropositive and 13 were SCV2 seronegative.

**Supplementary Figures**

**
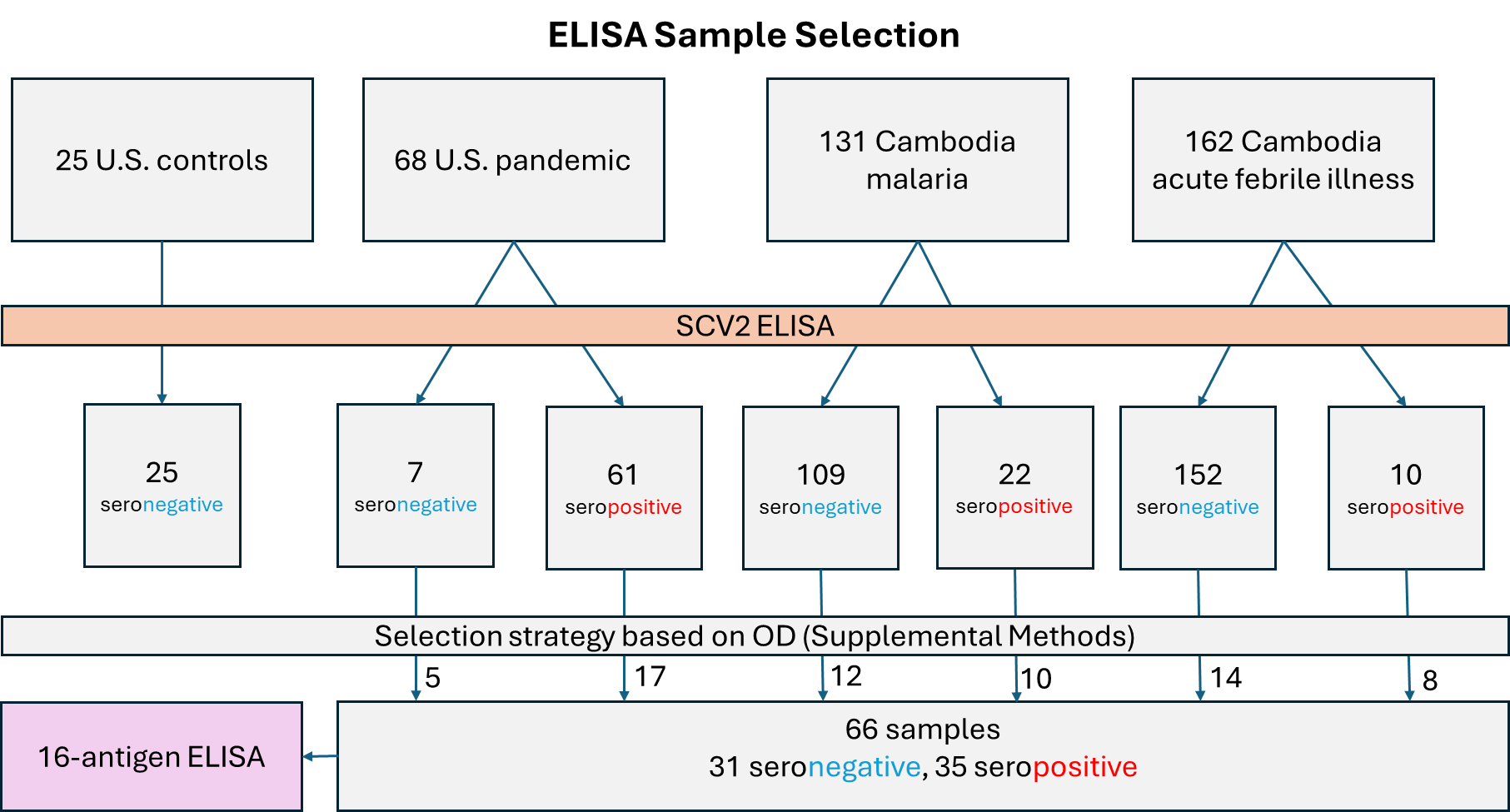
**

**Figure S1.** Sample selection for ELISA analyses. For initial studies (9-antigen ELISAs), 293 Cambodian samples, 68 U.S. pandemic samples, and 25 U.S. controls were used. From the U.S. pandemic samples and Cambodian samples, a total of 66 samples were selected to be used in the measurement of reactivity against an additional 7 antigens, for a total of 16 antigens.

**
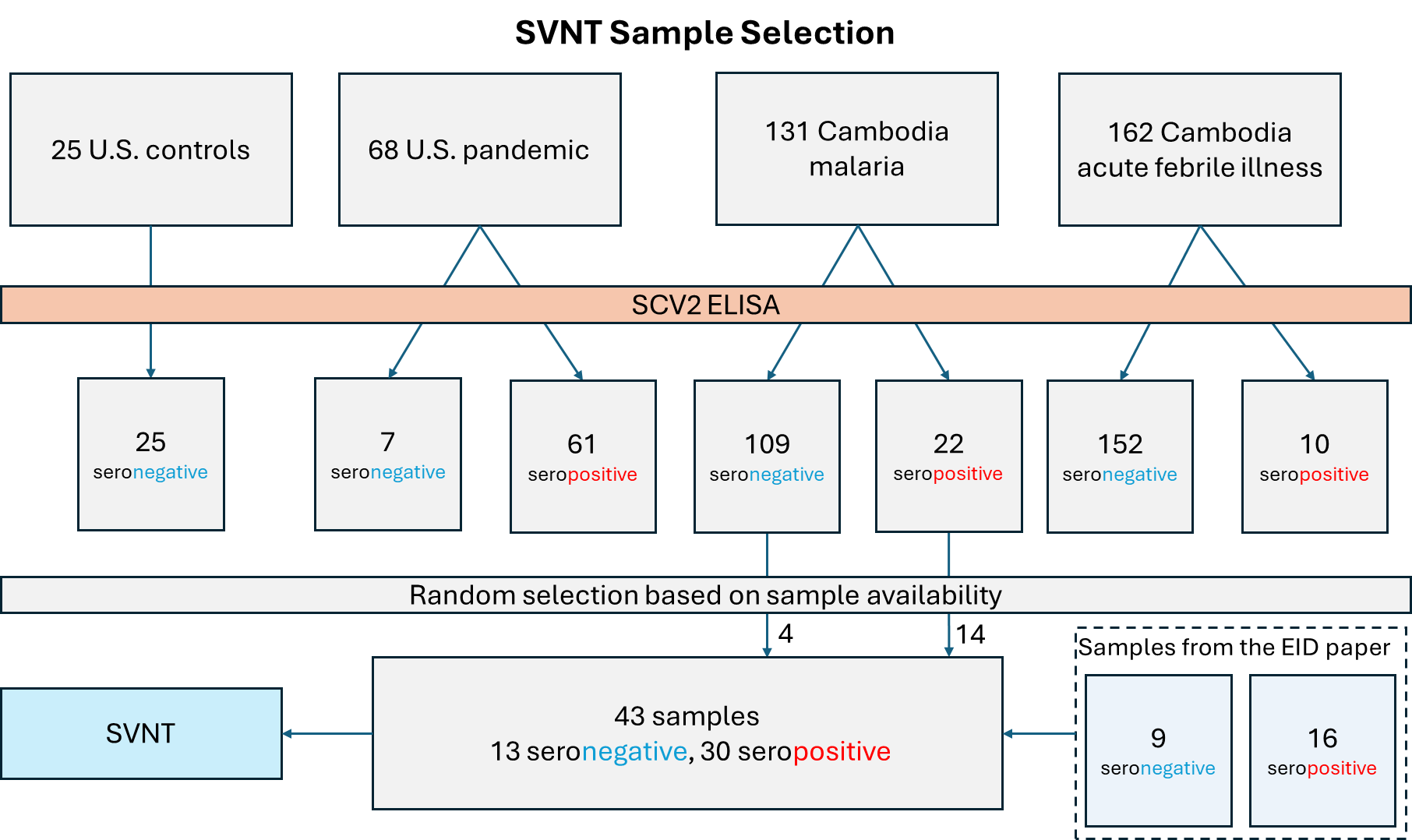
**

**Figure S2.** Sample selection for SVNT analyses. SVNT analysis was conducted on some of the samples used in initial studies (9-antigen ELISAs) from this paper based on sample availability, in addition to some samples that had been used in a previous publication.

**
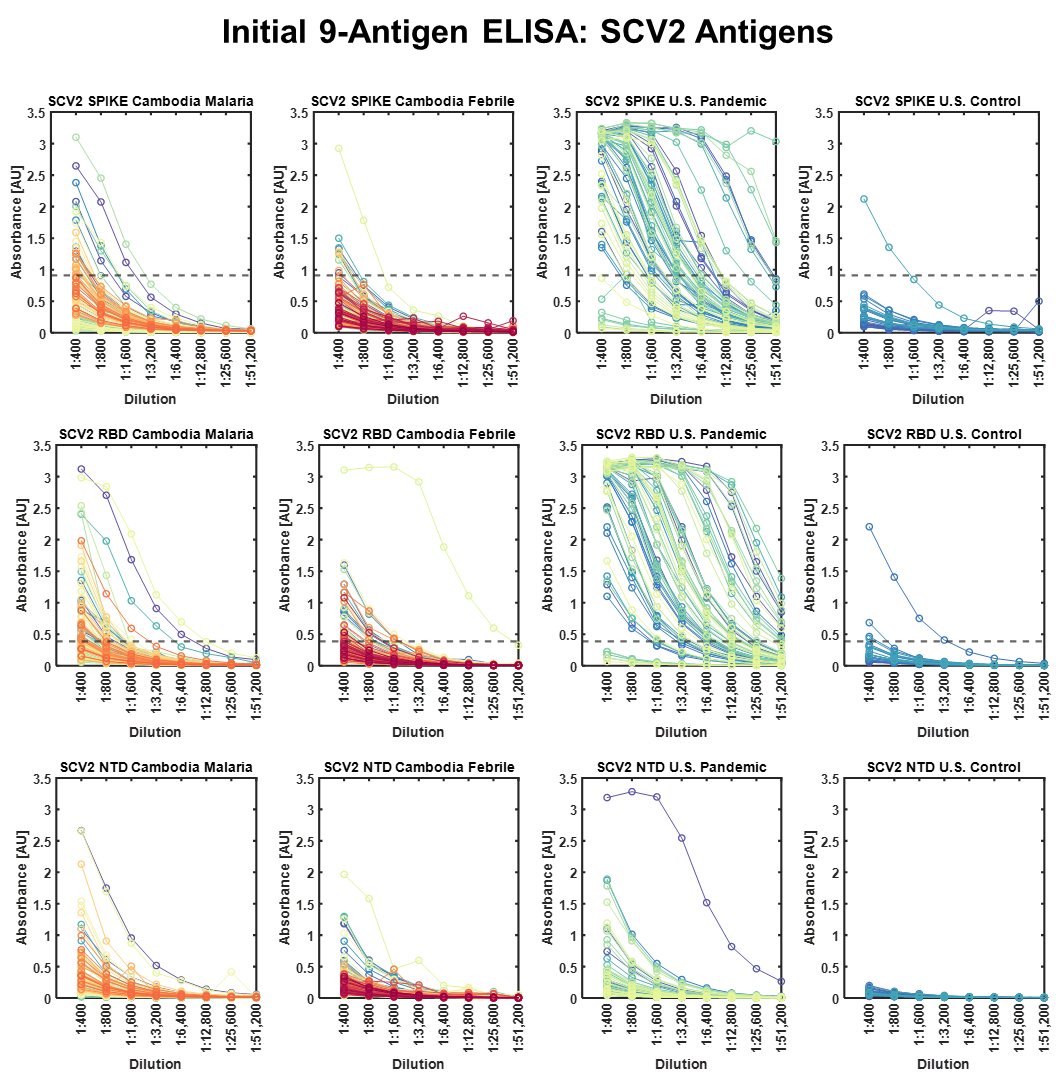
**

**Figure S3.** Titer curves measuring reactivity of Cambodia acute malaria (n=131), Cambodia acute febrile illness (n=168), U.S. pandemic (n=68), and U.S control (n=25) samples against SCV2 SPIKE, SCV2 RBD, and SCV2 NTD.


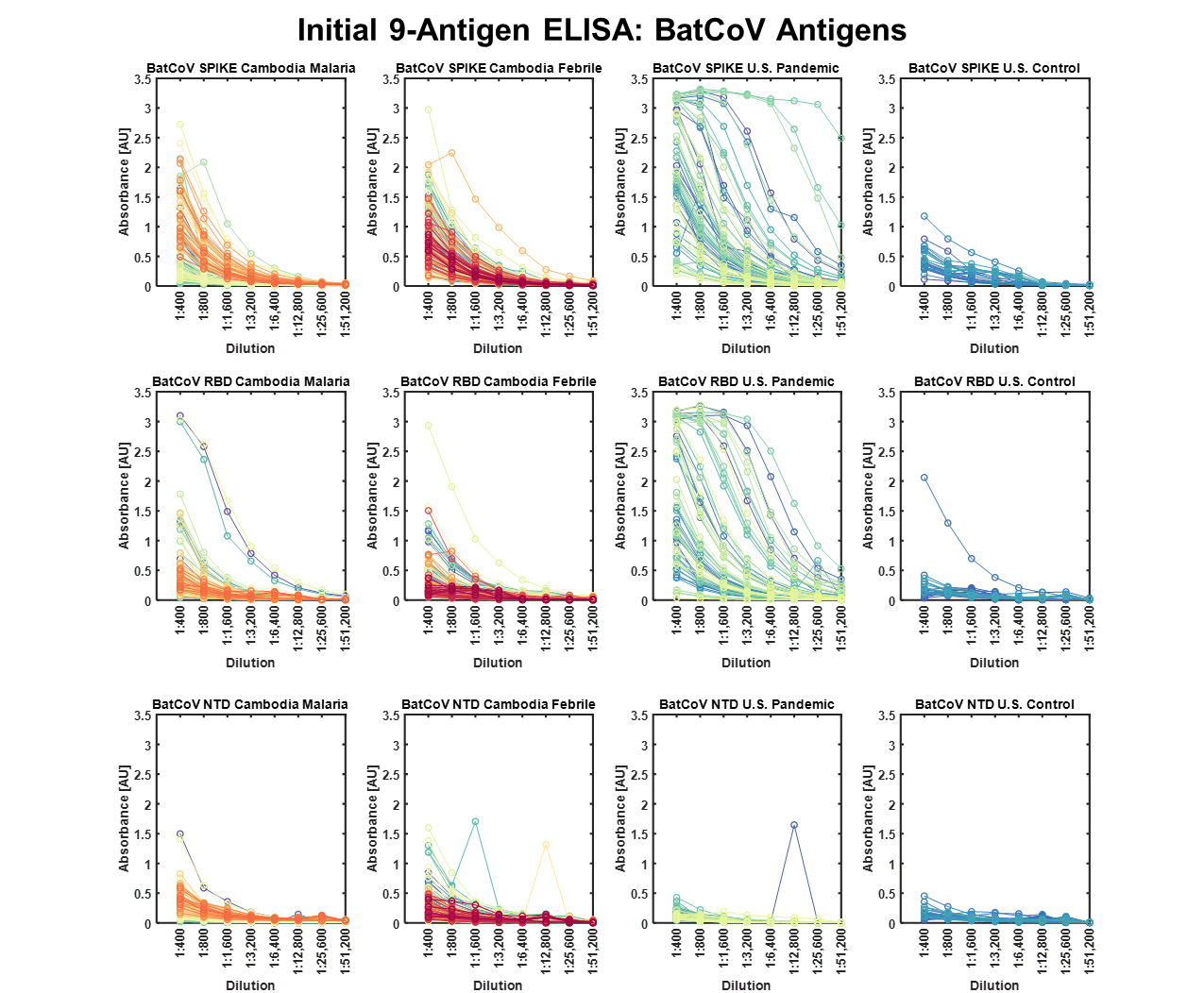


**Figure S4.** Titer curves measuring reactivity of Cambodia acute malaria (n=131), Cambodia acute febrile illness (n=168), U.S. pandemic (n=68), and U.S control (n=25) samples against BatCoV SPIKE, BatCoV RBD, and BatCoV NTD.

**
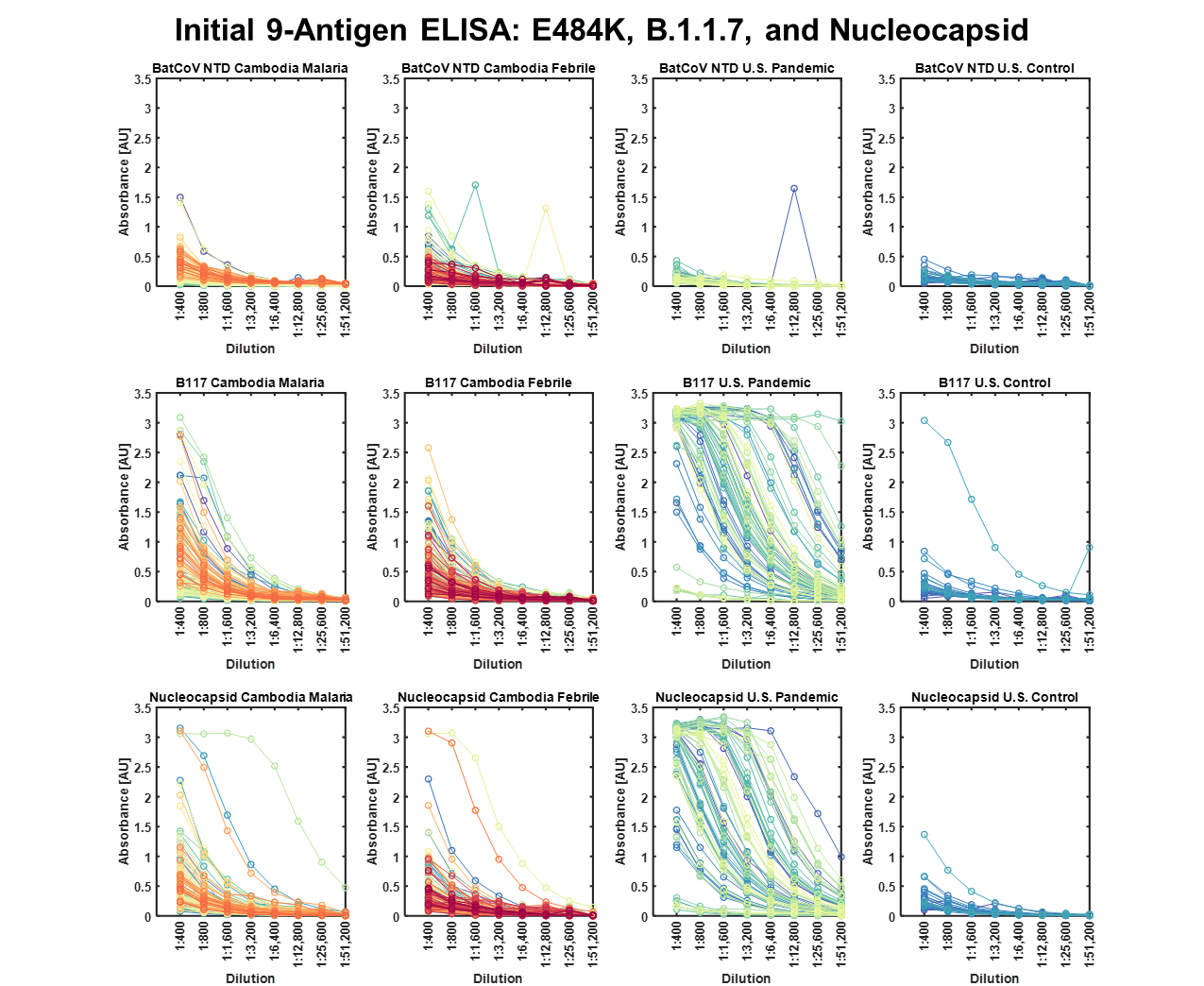
**

**Figure S5.** Titer curves measuring reactivity of Cambodia acute malaria (n=131), Cambodia acute febrile illness (n=168), U.S. pandemic (n=68), and U.S control (n=25) samples against E484K, B.1.1.7, and Nucleocapsid.

**
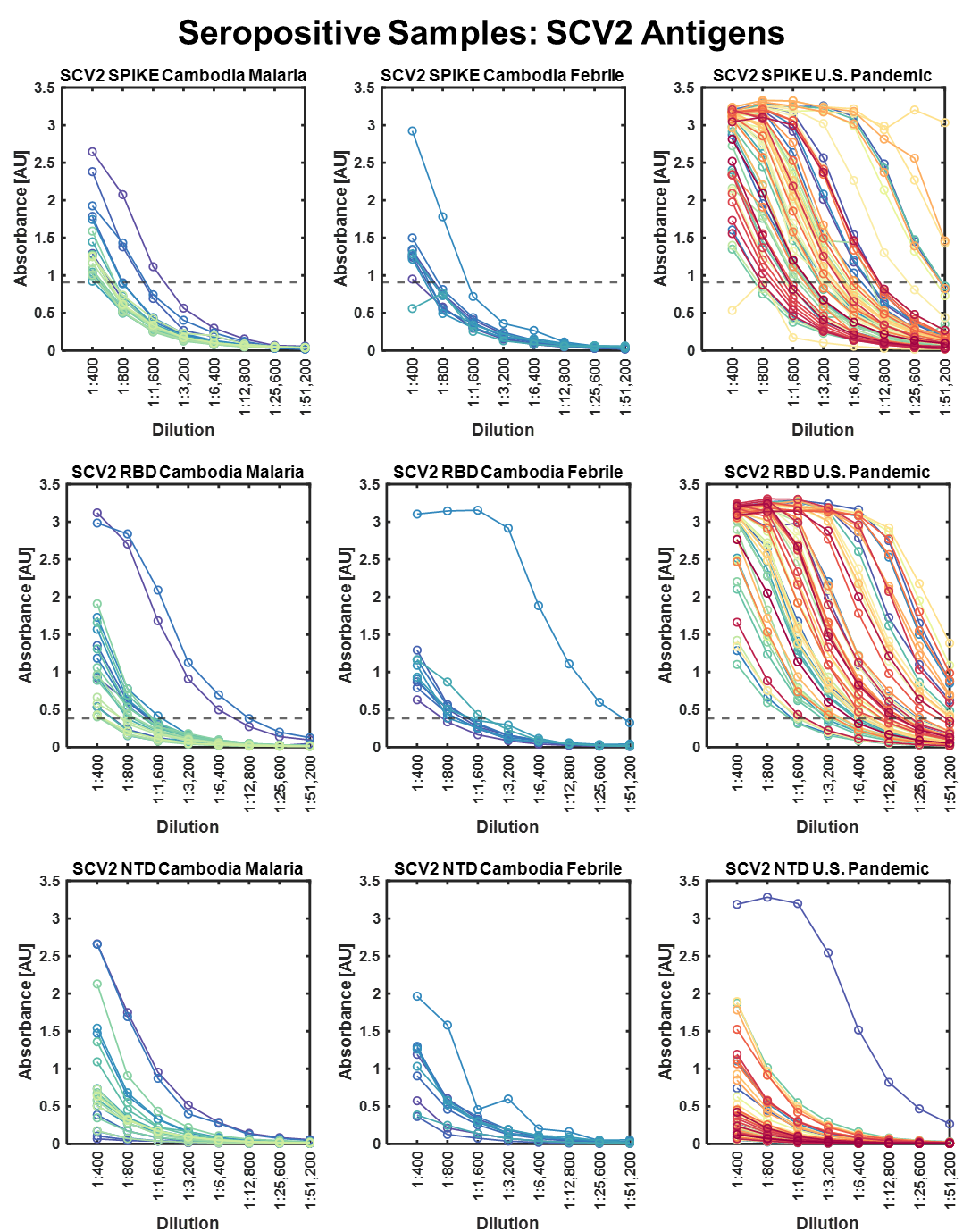
**

**Figure S6.** Titer curves measuring reactivity of seropositive Cambodia acute malaria (n=22), Cambodia acute febrile illness (n=10), and seropositive U.S. pandemic (n=61) samples against SCV2 SPIKE, SCV2 RBD, and SCV2 NTD

**
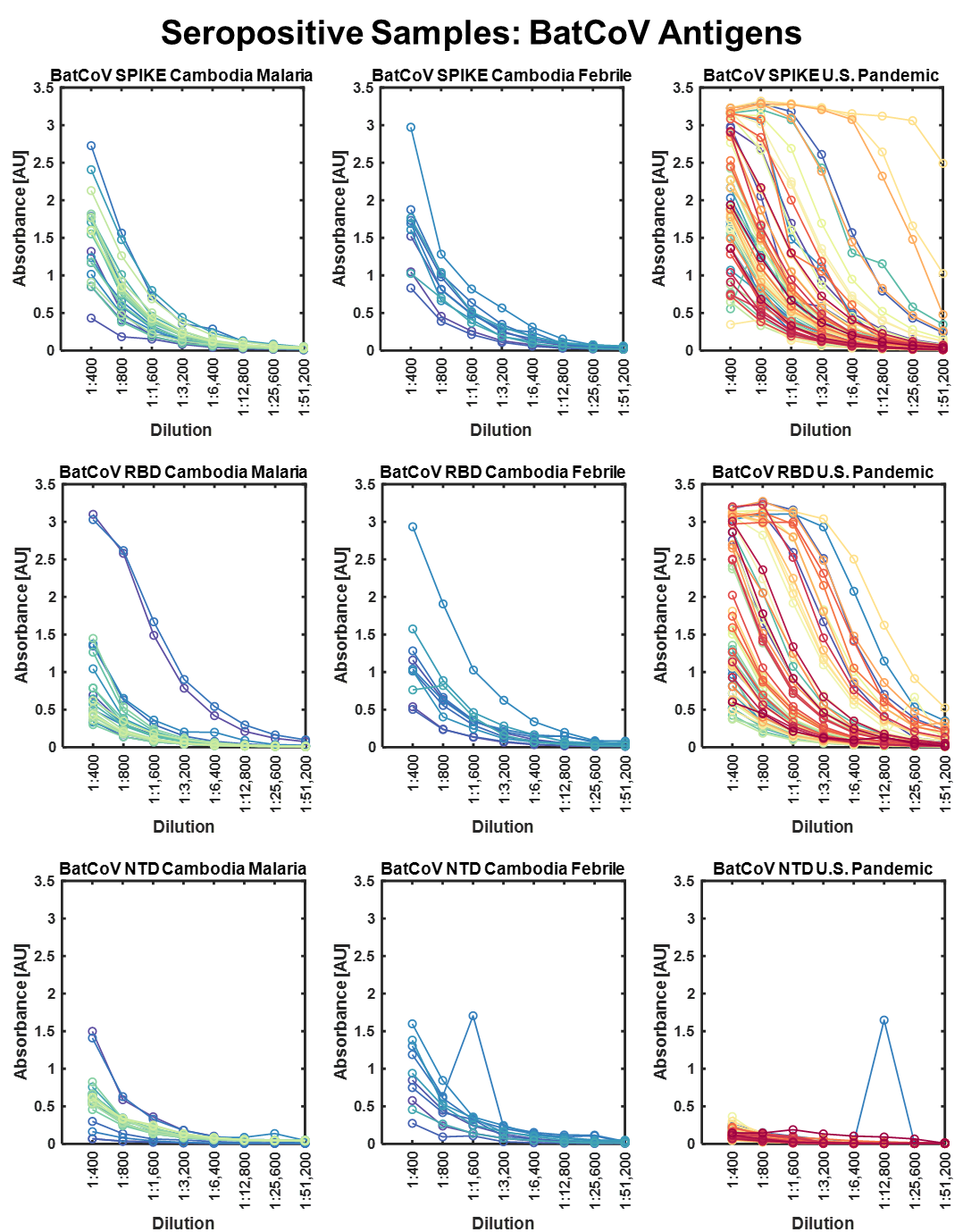
**

**Figure S7.** Titer curves measuring reactivity of seropositive Cambodia acute malaria (n=22), Cambodia acute febrile illness (n=10), and seropositive U.S. pandemic (n=61) samples against BatCoV SPIKE, BatCoV RBD, and BatCoV NTD.

**
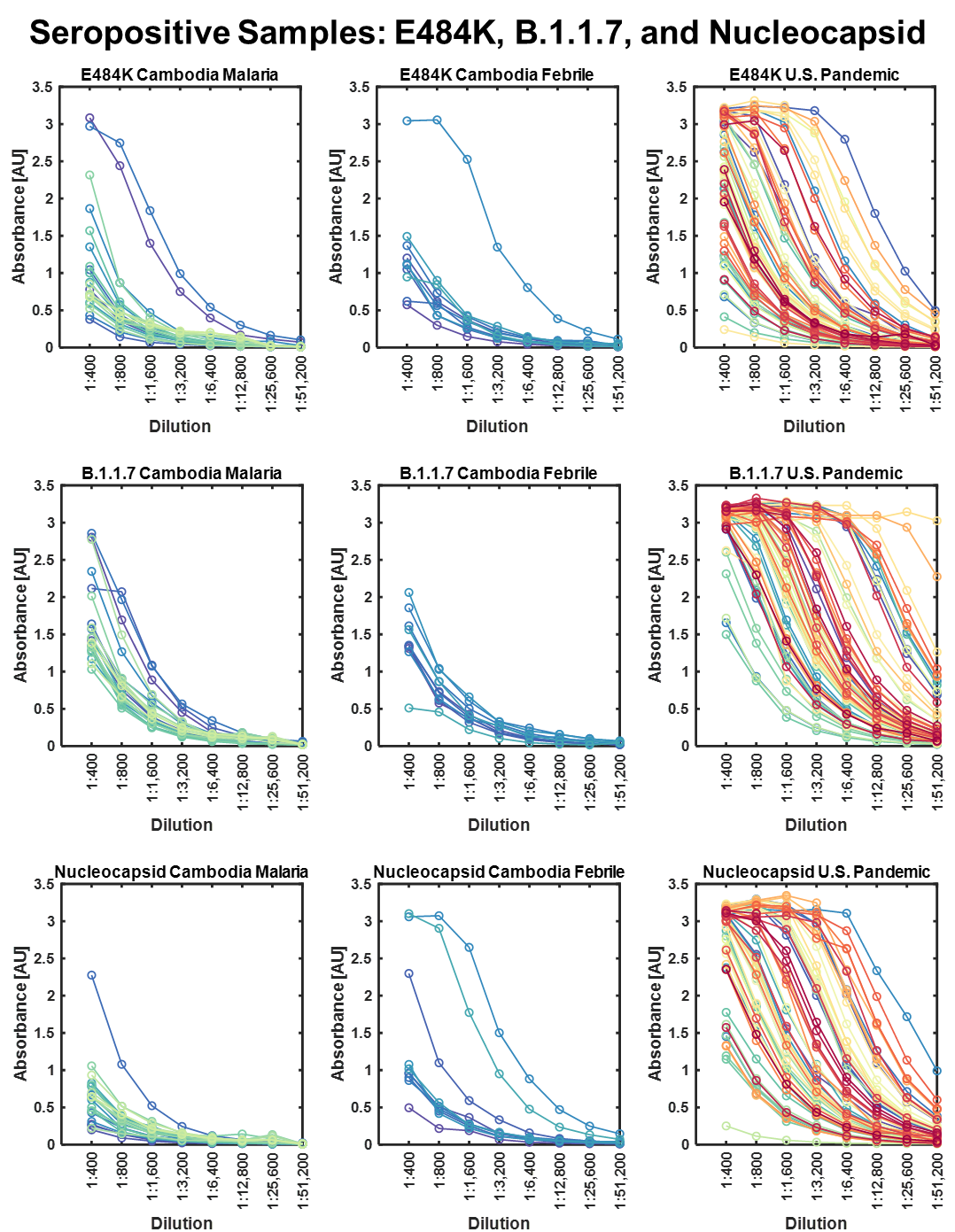
**

**Figure S8.** Titer curves measuring reactivity of seropositive Cambodia acute malaria (n=22), Cambodia acute febrile illness (n=10), and U.S. pandemic (n=61) samples against E484K, B.1.1.7, and Nucleocapsid.

**
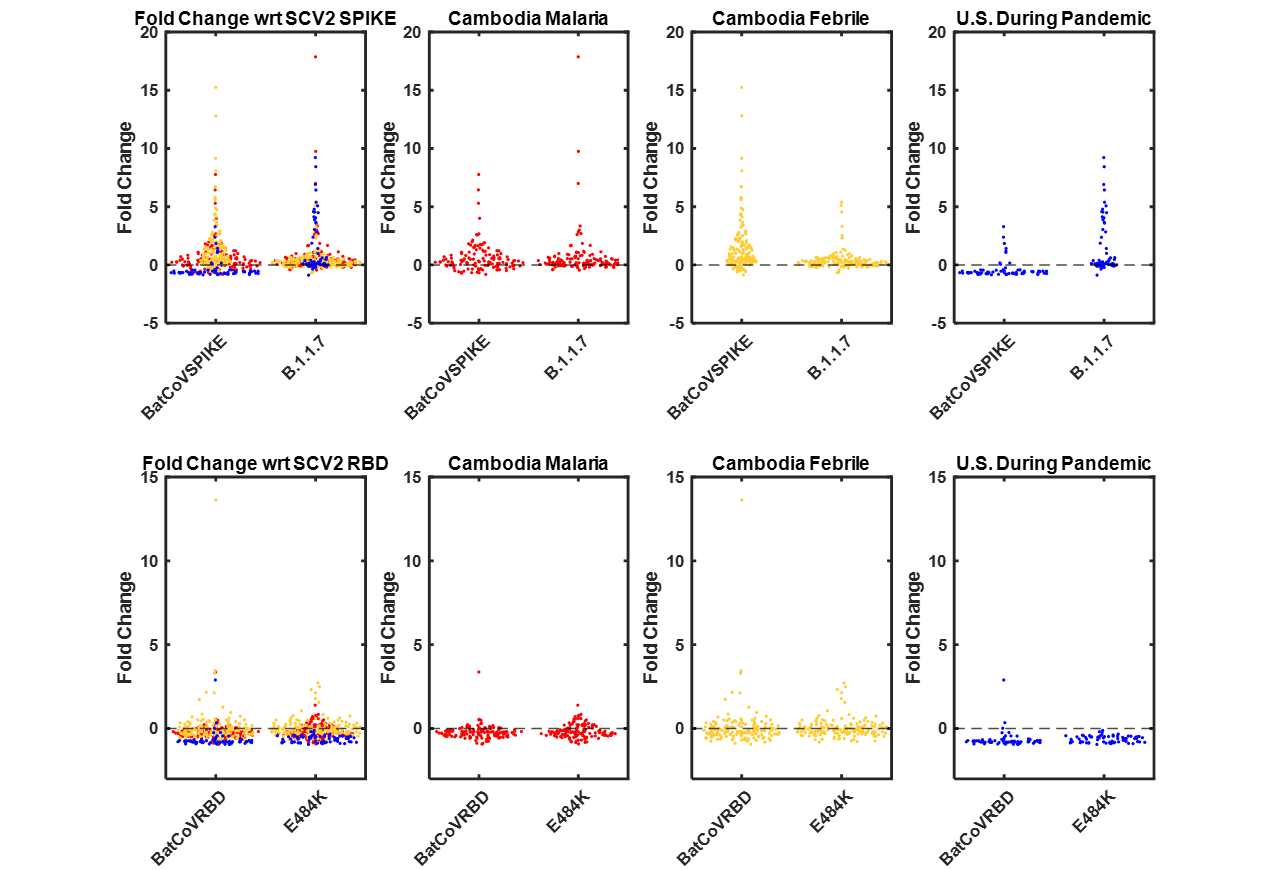
**

**Figure S9.** Fold change in reactivity of Cambodia acute malaria (n=131), Cambodia acute febrile illness (n=168), and U.S. pandemic (n=68) samples against BatCoV proteins and SCV2 variants compared to regular SCV2 antigens.

**
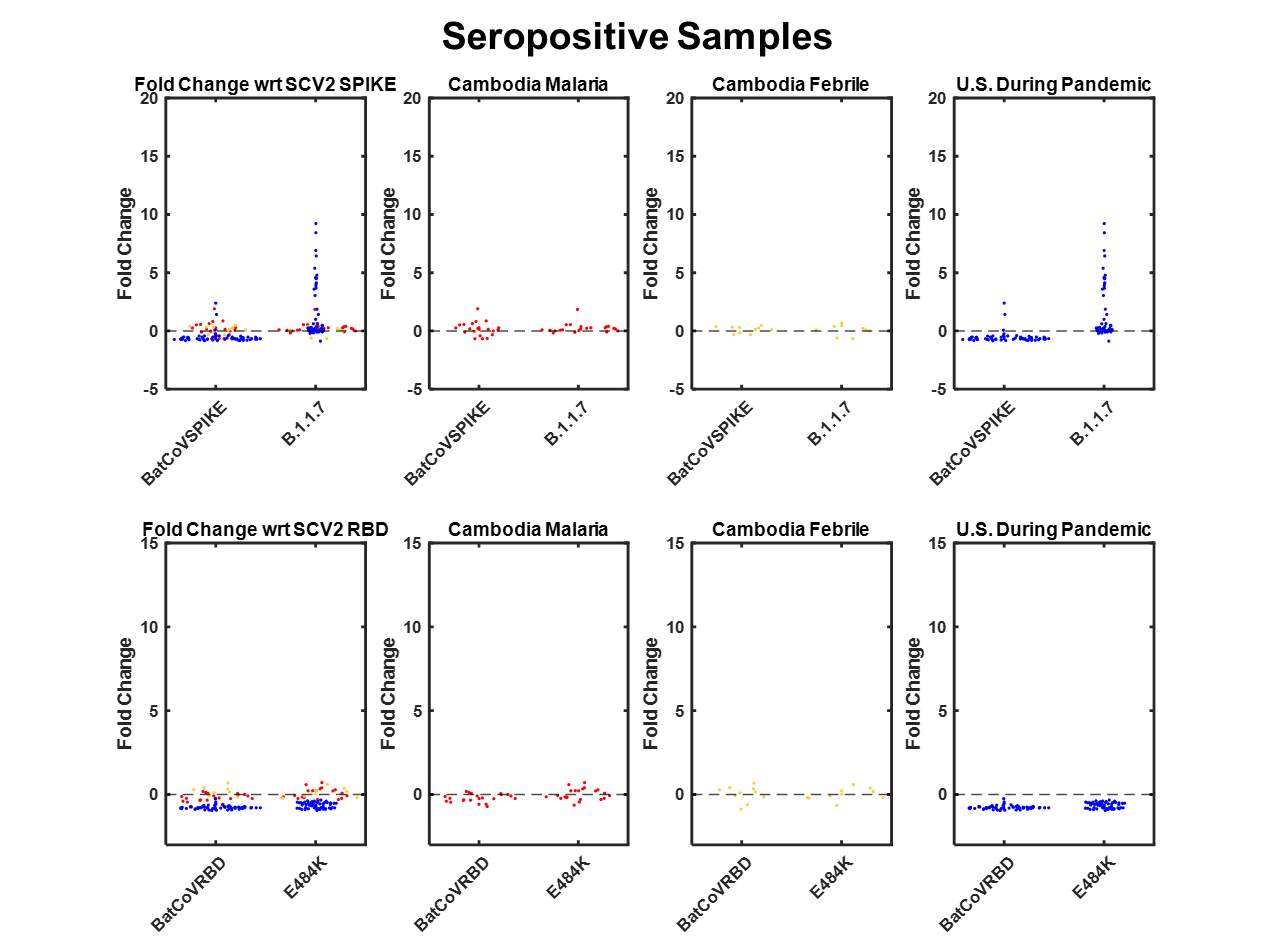
**

**Figure S10.** Fold change in reactivity of seropositive Cambodia acute malaria (n=22), Cambodia acute febrile illness (n=10), and U.S. pandemic (n=61) samples against BatCoV proteins and SCV2 variants compared to regular SCV2 antigens.

**
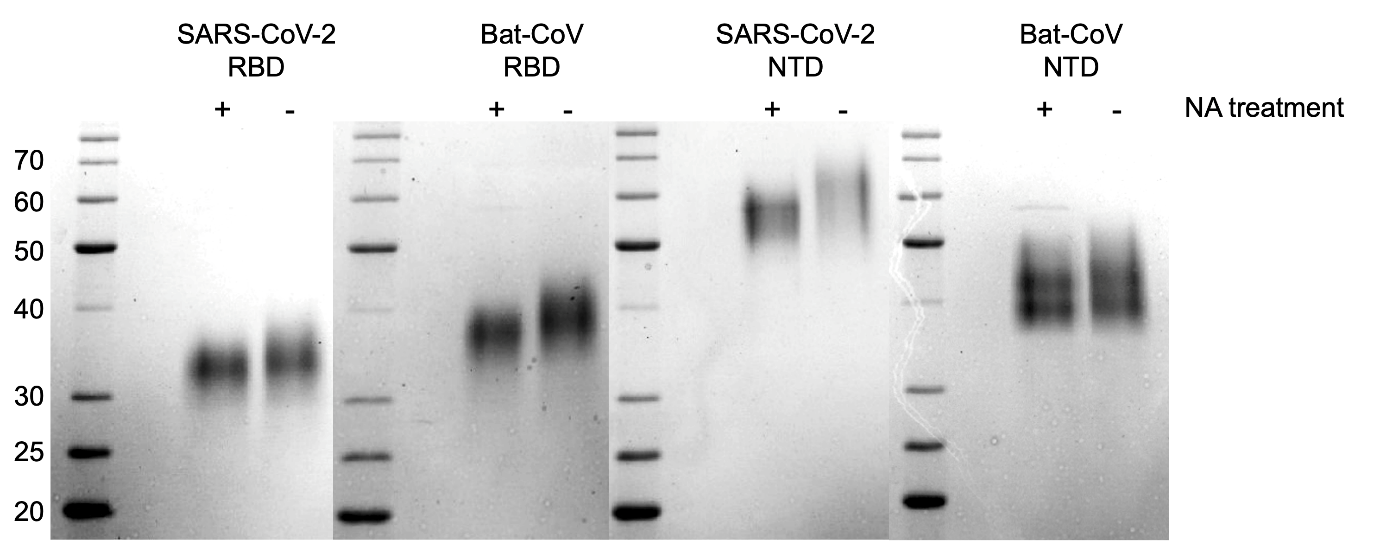
**

**Figure S11.** SDS-PAGE analysis of purified antigens before and after neuraminidase treatment. Pairs of samples are pre (-) and post (+) neuraminidase treatment, showing the reduction in size of the protein after removal of sialic acids from glycans. Molecular weight markers are shown in kilodaltons.


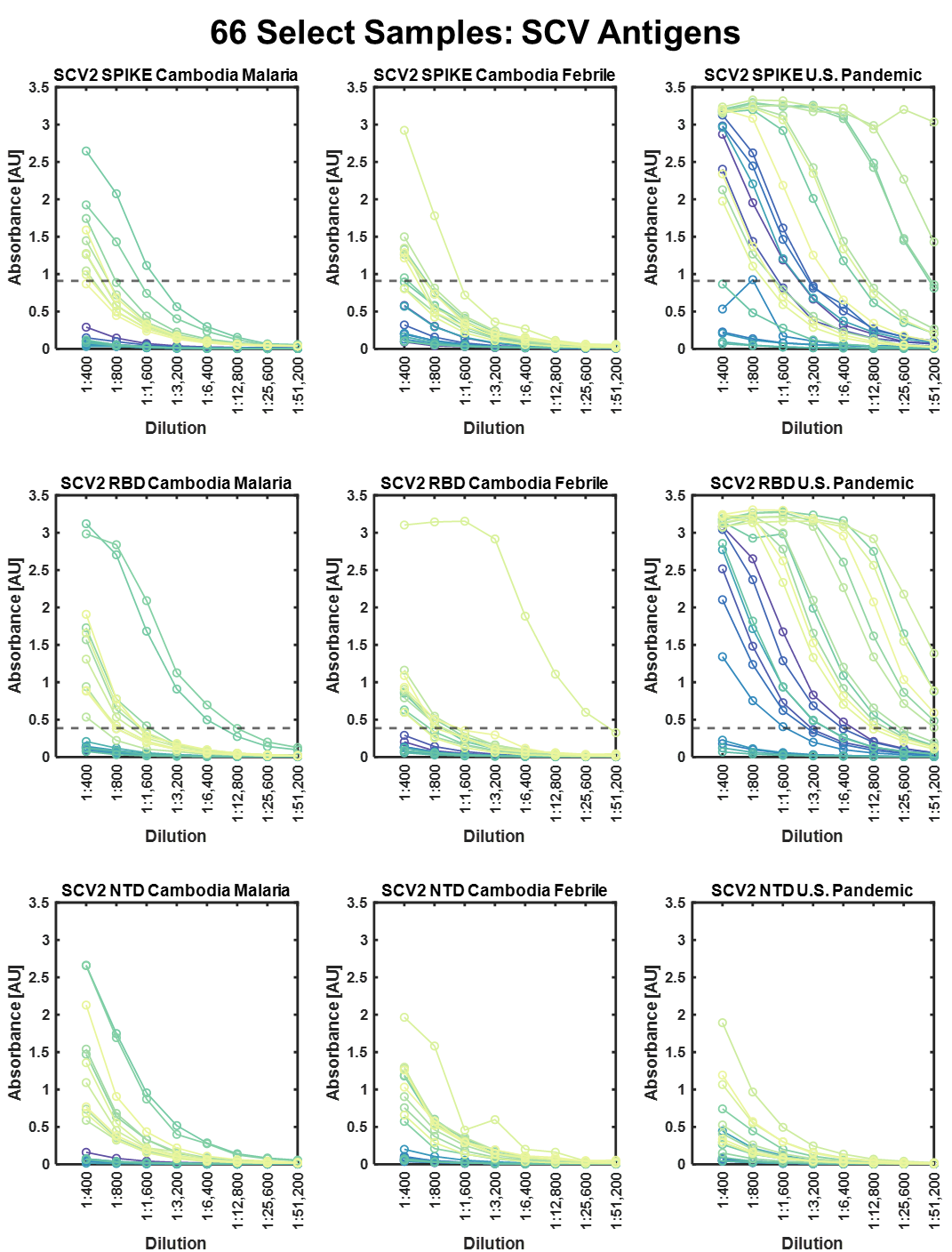


**Figure S12.** Titer curves measuring reactivity of select Cambodia acute malaria (n=22), select Cambodia acute febrile illness (n=22), select U.S. pandemic (n=22) samples against SCV2 SPIKE, SCV2 RBD, and SCV2 NTD.

**
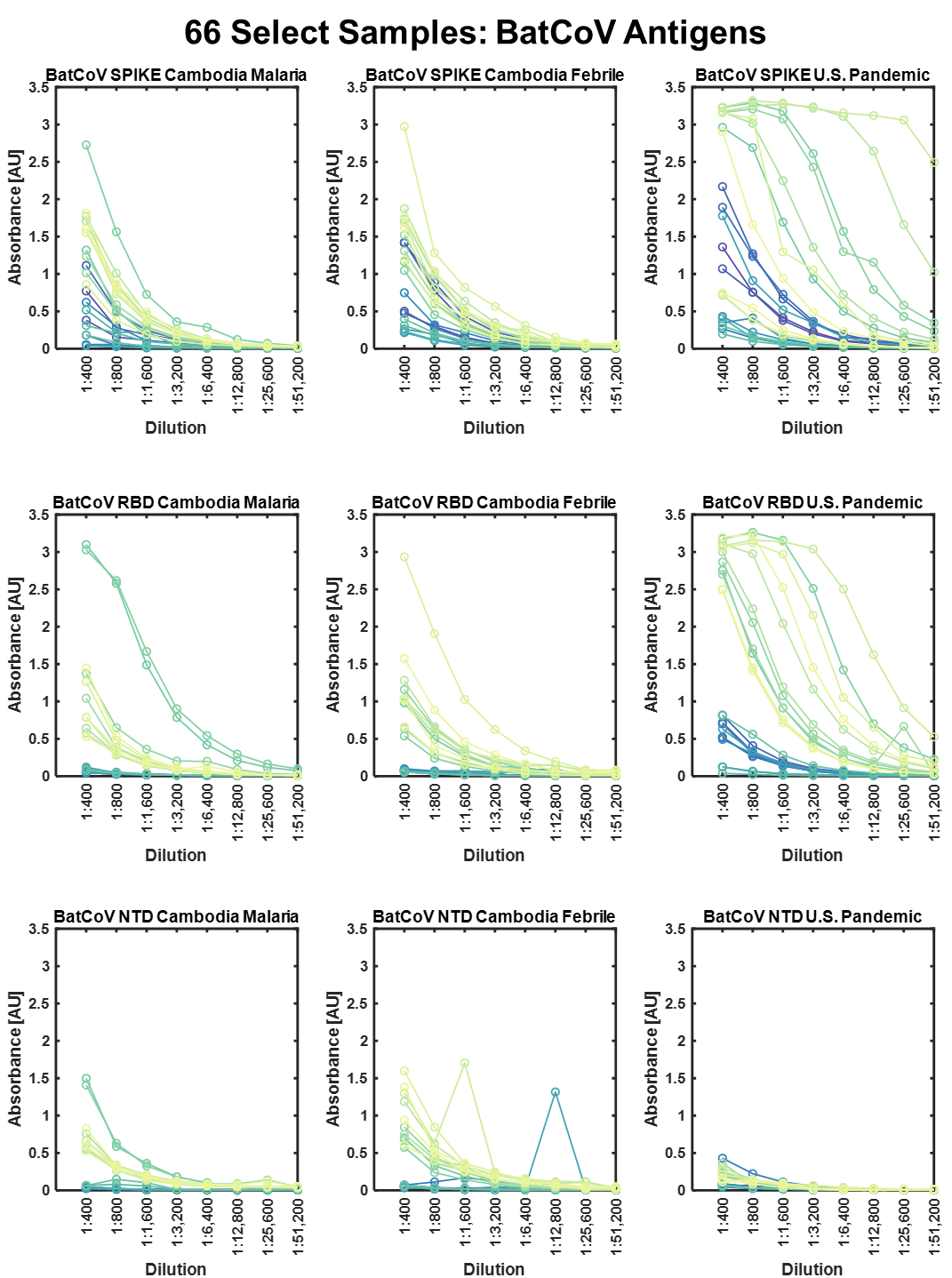
**

**Figure S13.** Titer curves measuring reactivity of select Cambodia acute malaria (n=22), select Cambodia acute febrile illness (n=22), select U.S. pandemic (n=22) samples against BatCoV SPIKE, BatCoV RBD, and BatCoV NTD.

**
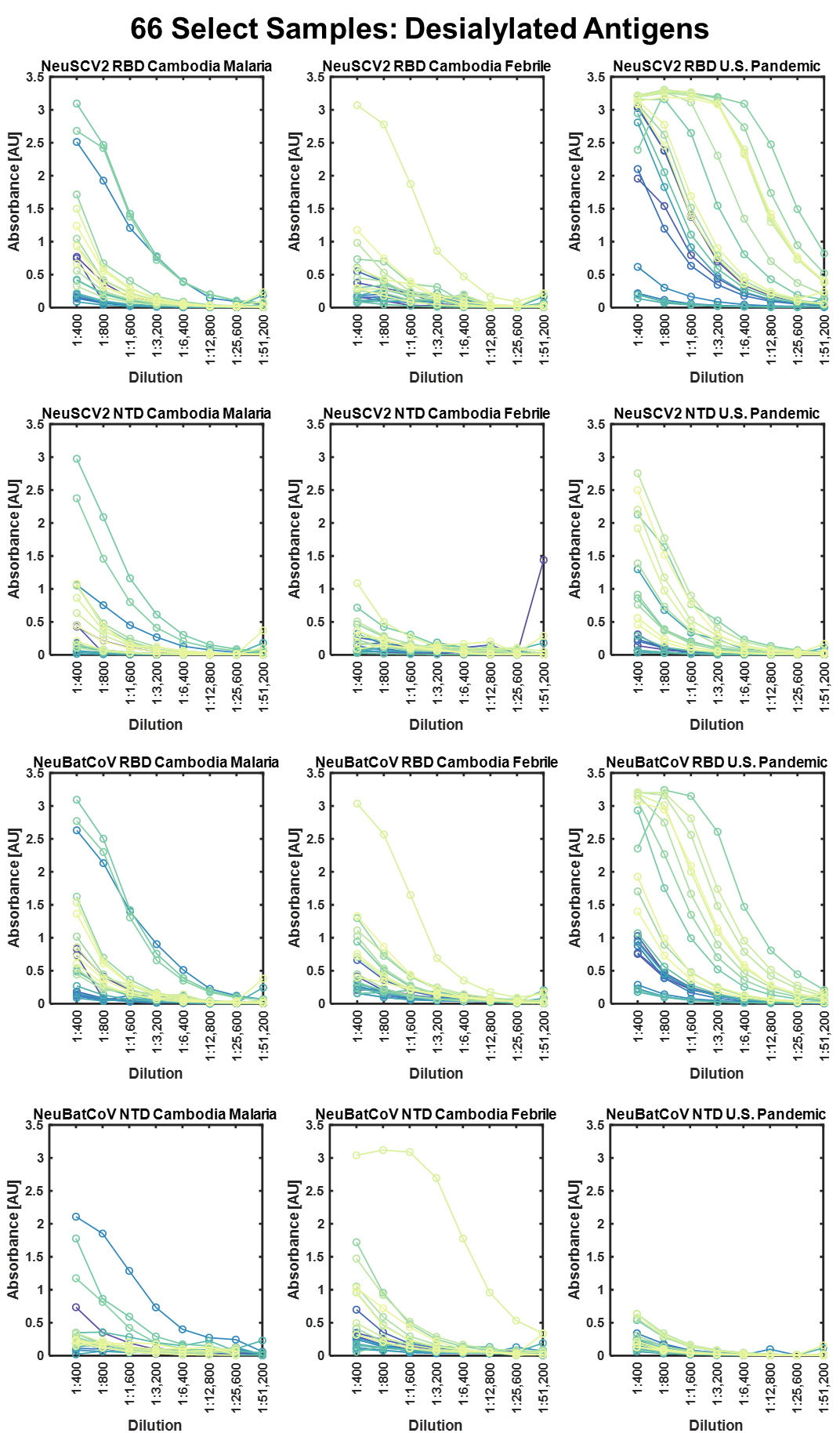
**

**Figure S14.** Titer curves measuring reactivity of select Cambodia acute malaria (n=22), select Cambodia acute febrile illness (n=22), select U.S. pandemic (n=22) samples against desialylated versions of SCV2 RBD, SCV2 NTD, BatCoV RBD, and BatCoV NTD.


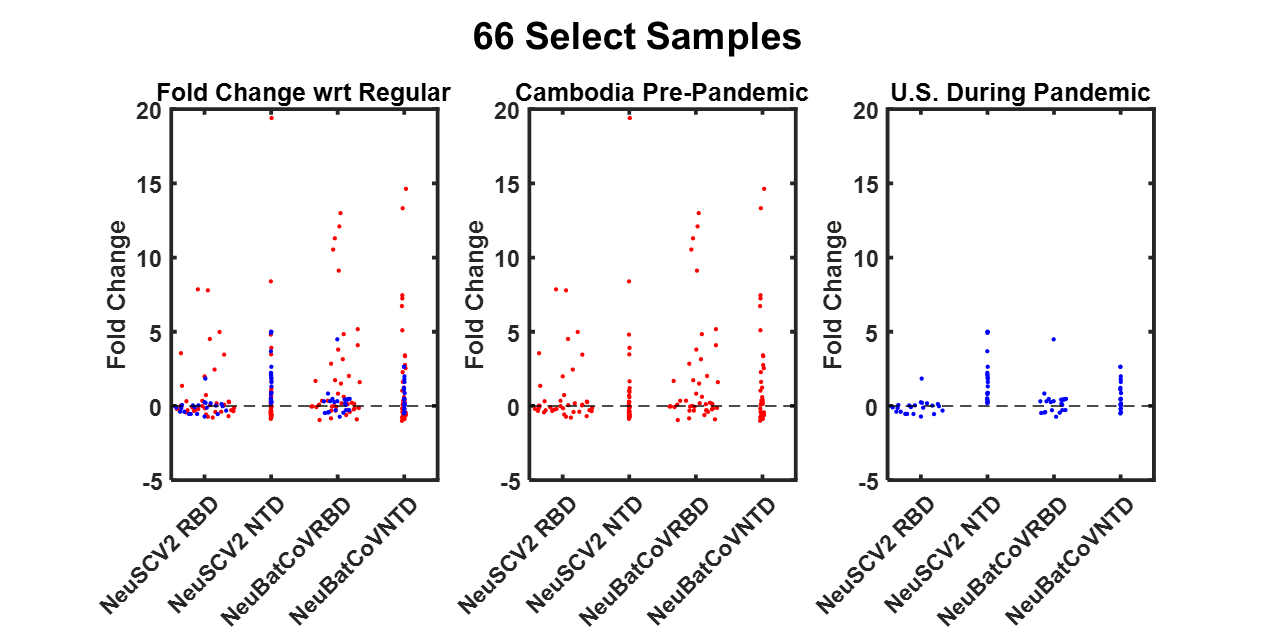


**Figure S15.** Fold change in reactivity of select Cambodia Pre-Pandemic (n=44) and select U.S. pandemic (n=22) samples against desialylated versions of SCV2 RBD, SCV2 NTD, BatCoV RBD, and BatCoV NTD compared to regular versions of each of these antigens.


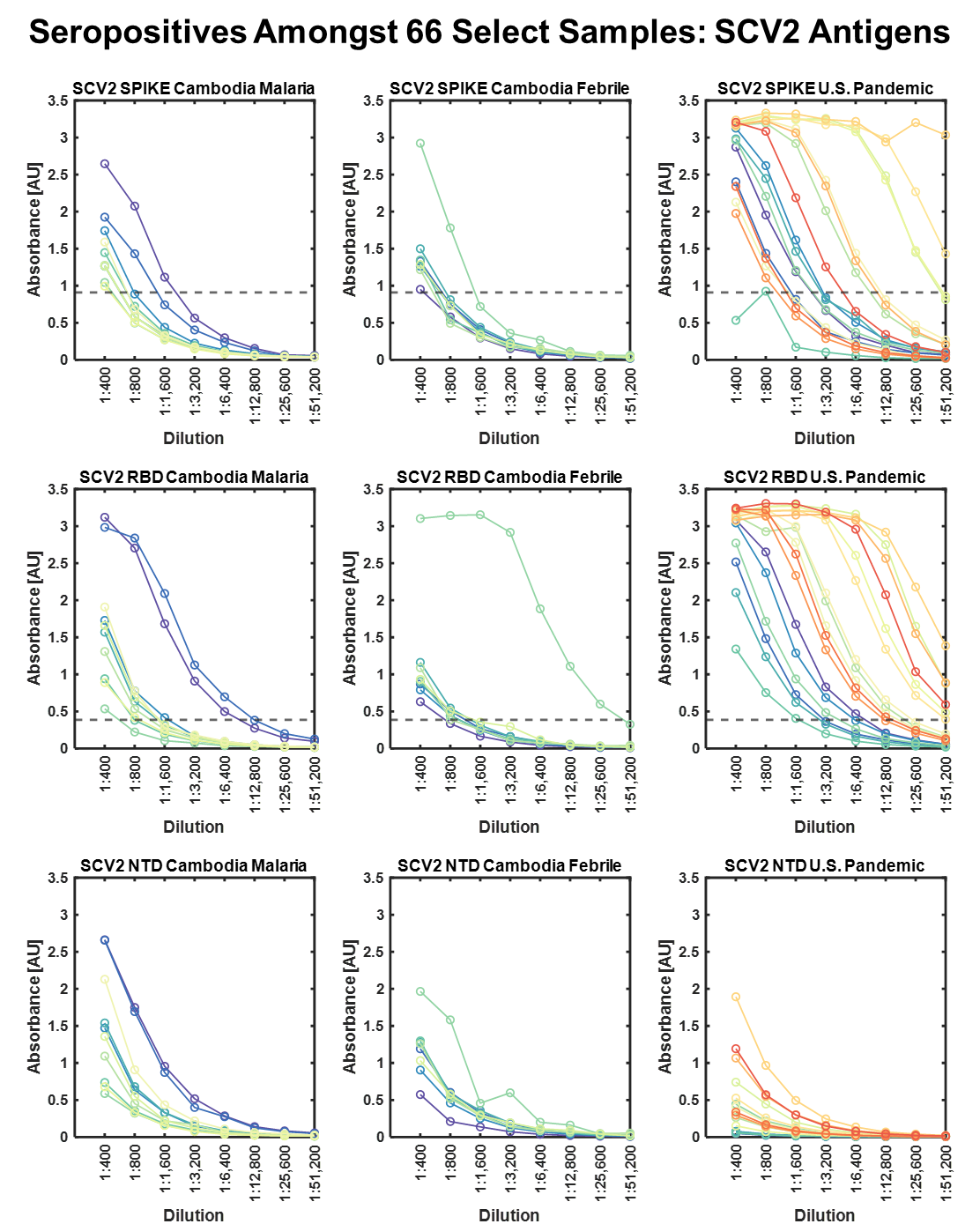


**Figure S16.** Titer curves measuring reactivity of select seropositive Cambodia Pre-Pandemic (n=18) and select seropositive U.S. pandemic (n=17) samples against SCV2 SPIKE, SCV2 RBD, and SCV2 NTD.


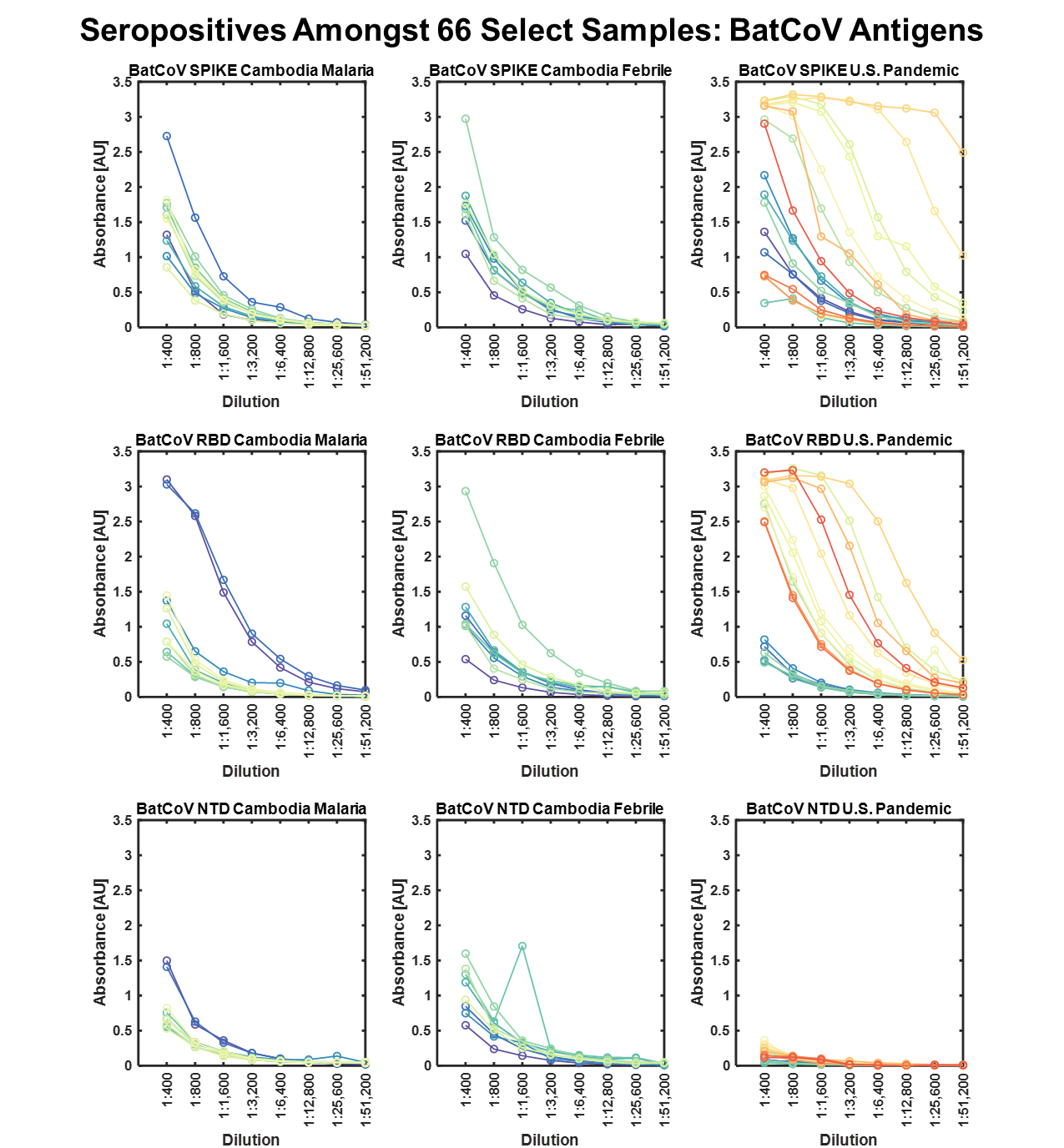


**Figure S17.** Titer curves measuring reactivity of select seropositive Cambodia Pre-Pandemic (n=18) and select seropositive U.S. pandemic (n=17) samples against BatCoV SPIKE, BatCoV RBD, and BatCoV NTD.


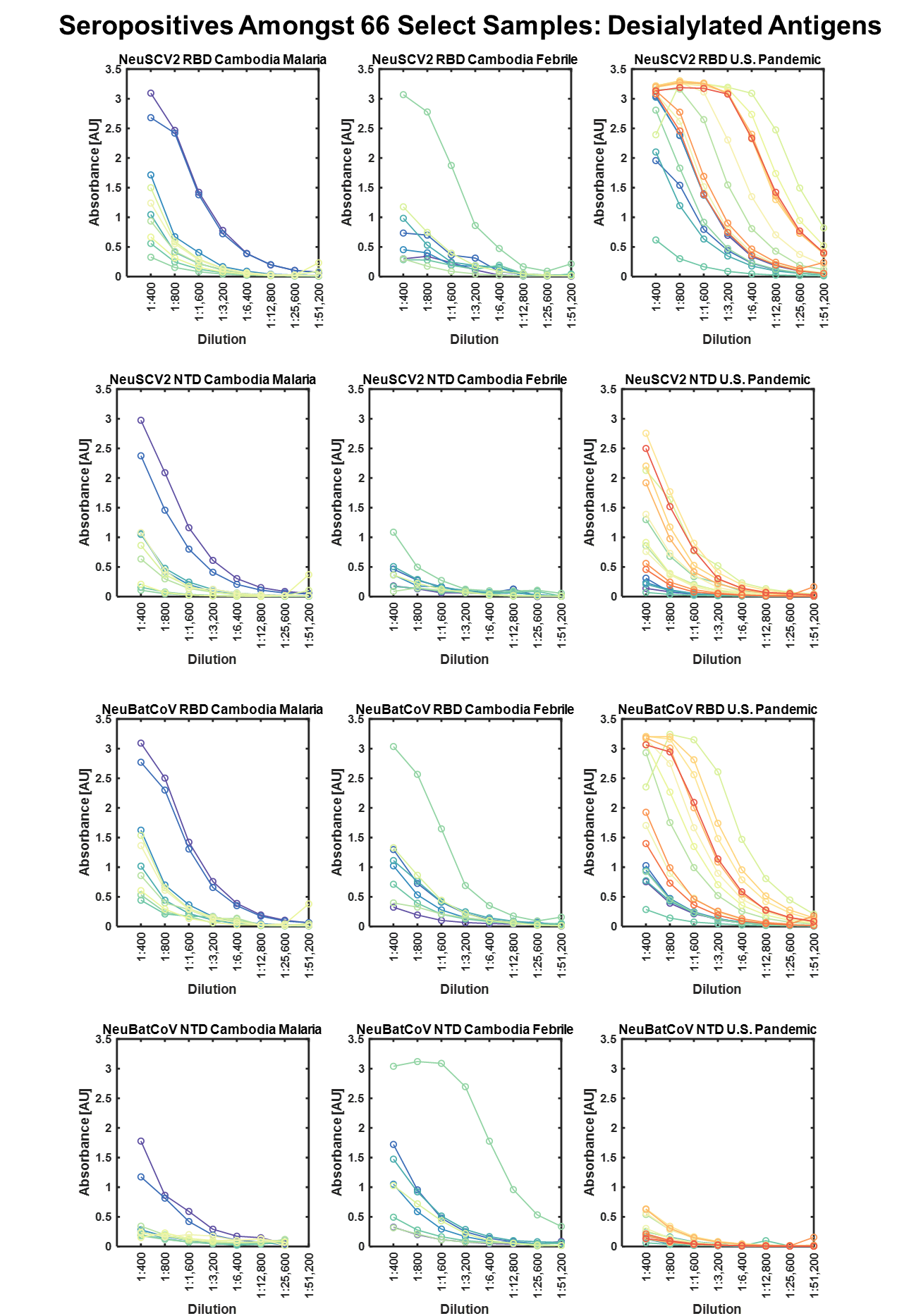


**Figure S18.** Titer curves measuring reactivity of select seropositive Cambodia Pre-Pandemic (n=18) and select seropositive U.S. pandemic (n=17) samples against desialylated versions of SCV2 RBD, SCV2 NTD, BatCoV RBD, and BatCoV NTD.


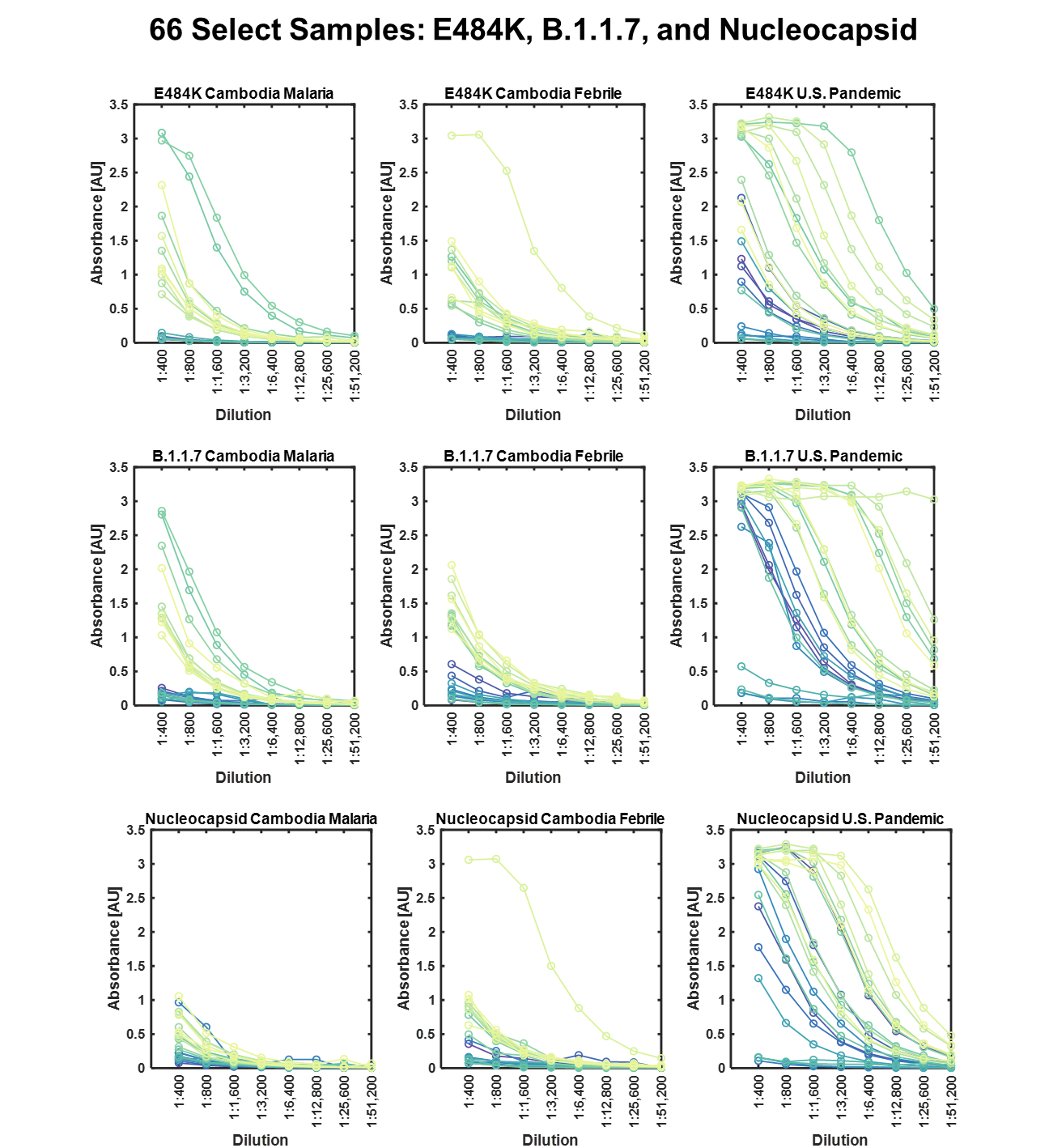


**Figure S19.** Titer curves measuring reactivity of select Cambodia acute malaria (n=22), select Cambodia acute febrile illness (n=22), and select U.S. pandemic (n=22) samples against SCV2 variants E484K and B.1.1.7, as well as Nucleocapsid.


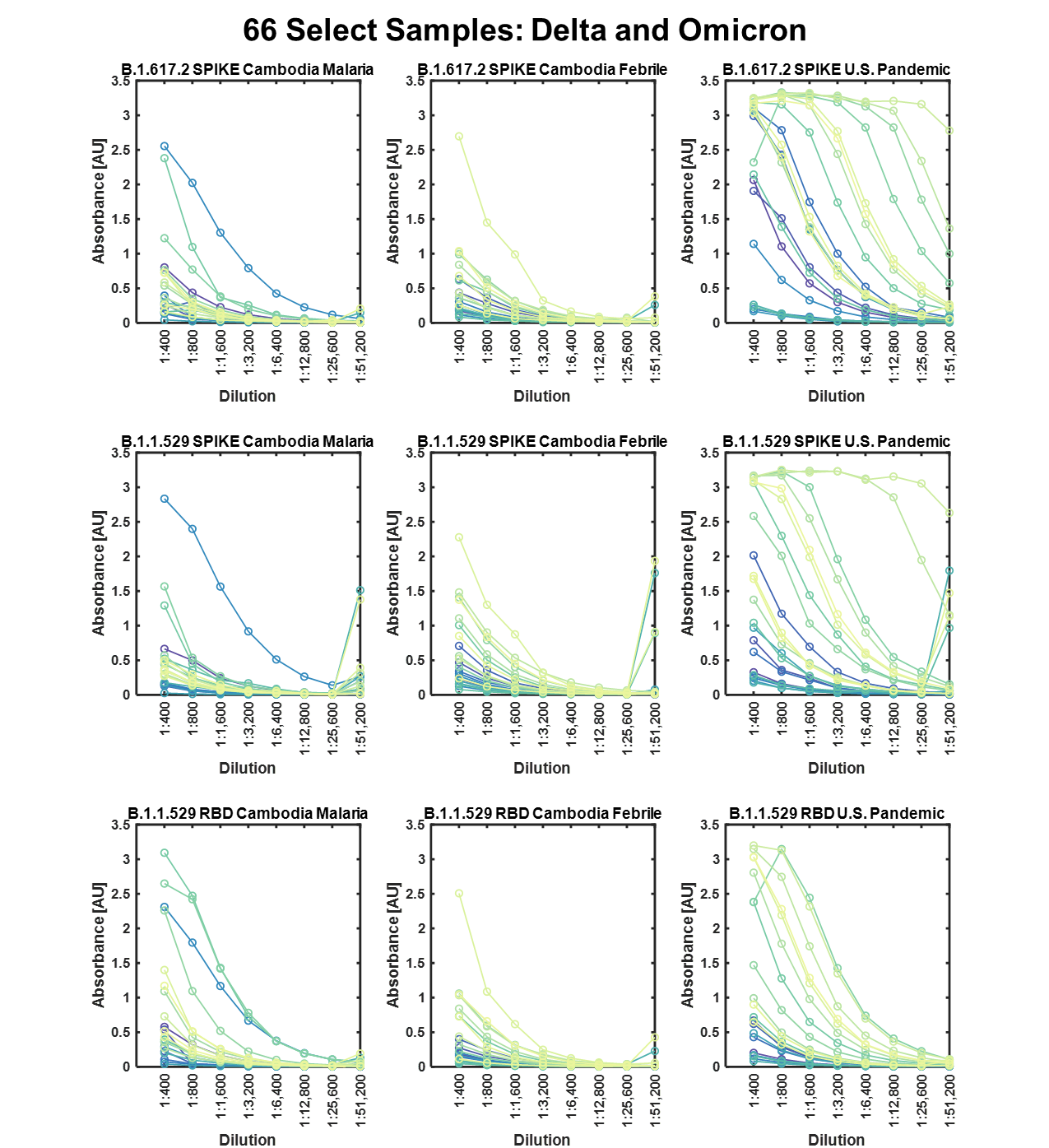


**Figure S20.** Titer curves measuring reactivity of select Cambodia acute malaria (n=22), select Cambodia acute febrile illness (n=22), and select U.S. pandemic (n=22) samples against SCV2 variants B.1.617.2 and B.1.1.529.


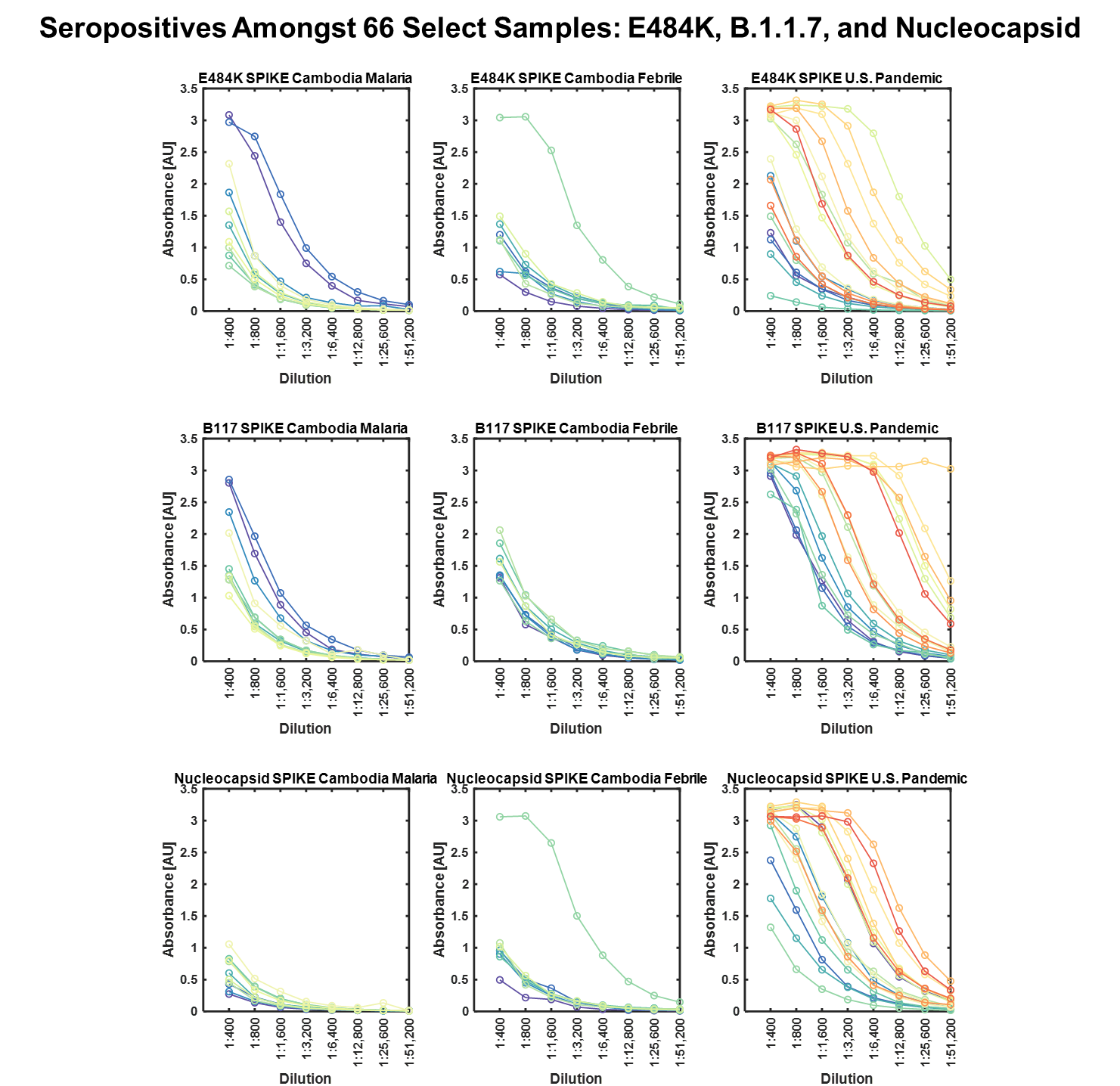


**Figure S21.** Titer curves measuring reactivity of select seropositive Cambodia Pre-Pandemic (n=18) and select seropositive U.S. pandemic (n=17) samples against SCV2 variants E484K and B.1.1.7, as well as Nucleocapsid.


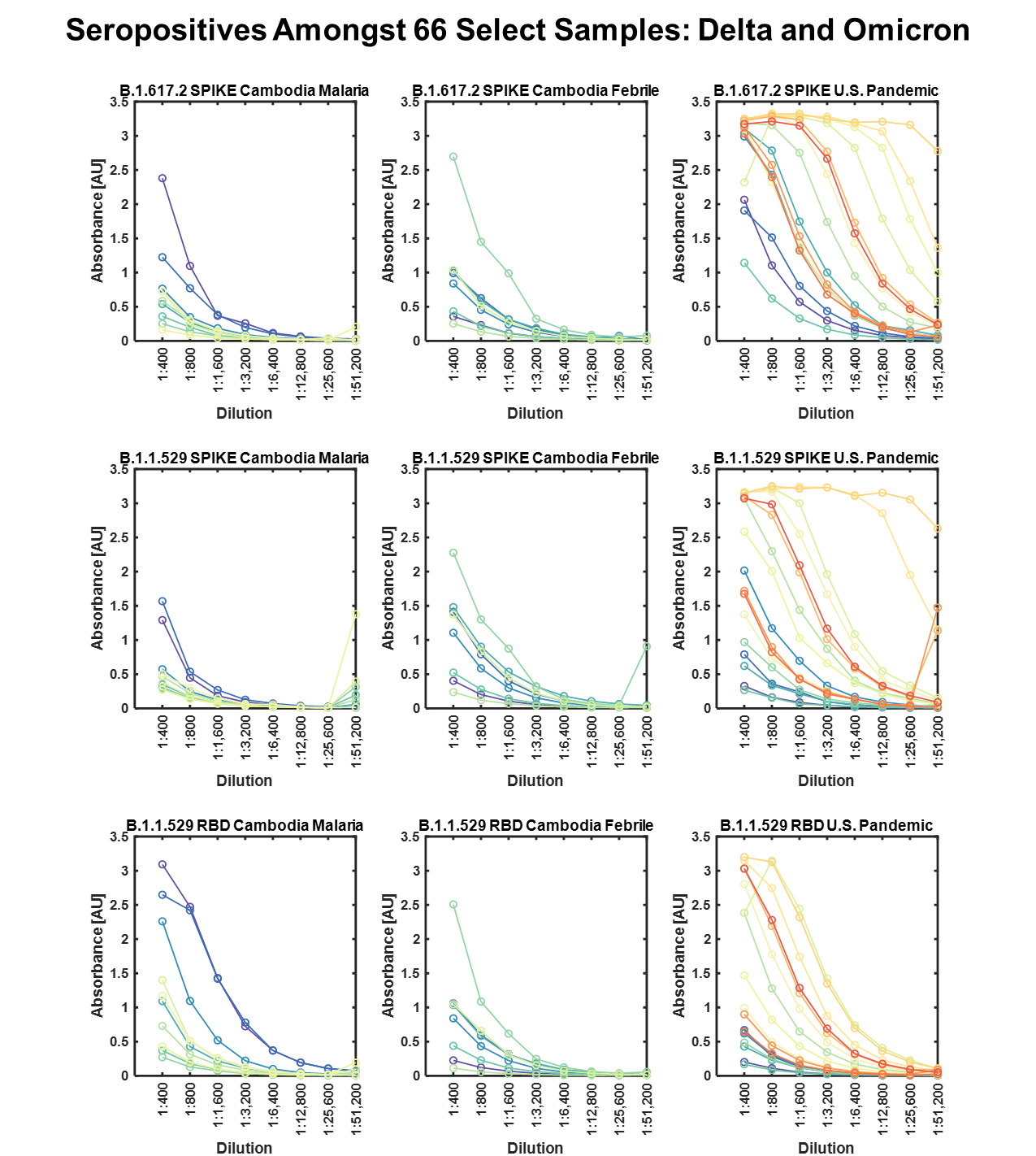


**Figure S22.** Titer curves measuring reactivity of select seropositive Cambodia Pre-Pandemic (n=18) and select seropositive U.S. pandemic (n=17) samples against SCV2 variants B.1.617.2 and B.1.1.529.


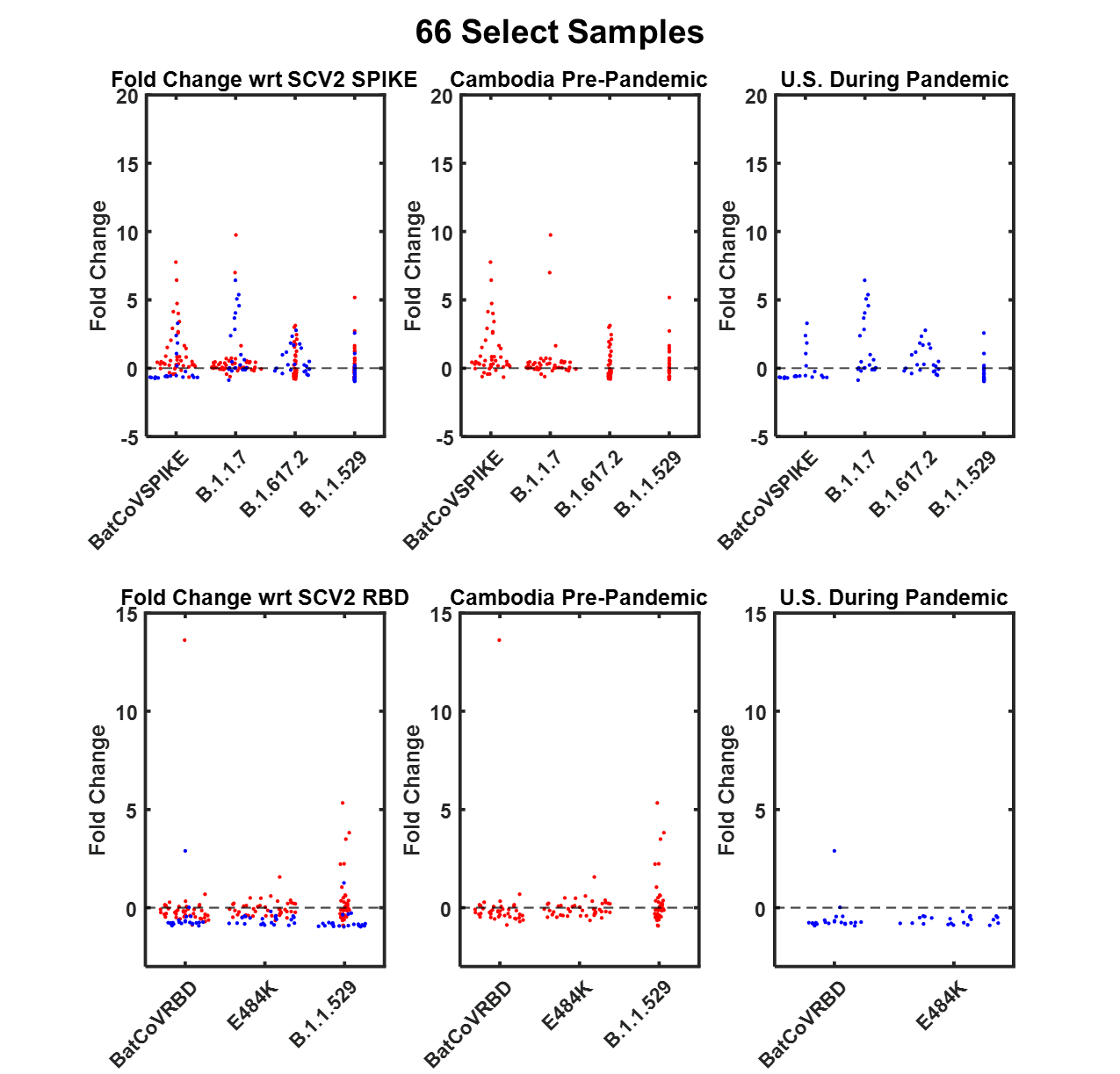


**Figure S23.** Fold change in reactivity of select Cambodia Pre-Pandemic (n=44) and select U.S. pandemic (n=22) samples against BatCoV proteins and SCV2 variants compared to SCV2 SPIKE and SCV2 RBD reactivity.

**Table S2.** Betacoronavirus ELISA values for 43 samples selected for SVNT.

| ID | Study | Normalized OD | | | | | | SCV2 serostatus |
| --- | --- | --- | --- | --- | --- | --- | --- | --- |
|  |  | SCV2 Spike | SCV2 RBD | SCV2 NTD | BatCoV Spike | BatCoV RBD | BatCoV NTD |  |
| EID115 | Manning et al | 1103.4 | 195.0 | 97.6 | 421.6 | 153.8 | 64.8 | Positive |
| EID140 | Manning et al | 1240.2 | 1224.6 | 1192.4 | 1197.0 | 1229.4 | 825.4 | Positive |
| EID425 | Manning et al | 310.6 | 165.6 | 65.0 | 340.4 | 206.2 | 57.0 | Negative |
| EID464 | Manning et al | 166.0 | 241.8 | 84.8 | 239.4 | 307.8 | 96.6 | Negative |
| EID860 | Manning et al | 1231.0 | 166.4 | 140.0 | 933.8 | 143.2 | 117.4 | Positive |
| EID874 | Manning et al | 371.6 | 369.2 | 316.8 | 341.8 | 394.4 | 231.0 | Positive |
| EID876 | Manning et al | 1142.2 | 874.0 | 687.6 | 620.6 | 1053.8 | 289.0 | Positive |
| KH001-035 | Manning et al | 544.8 | 1049.6 | 486.4 | 321.8 | 973.2 | 165.0 | Positive |
| KH001-043 | Manning et al | 373.0 | 400.0 | 225.2 | 344.8 | 552.8 | 169.4 | Positive |
| KH002-001 | Manning et al | 219.8 | 429.2 | 90.8 | 262.4 | 255.0 | 47.2 | Negative |
| KH002-004 | Manning et al | 1287.4 | 1174.0 | 986.4 | 1278.2 | 1227.6 | 258.4 | Positive |
| KH002-006 | Manning et al | 48.2 | 36.0 | 31.0 | 67.2 | 71.8 | 32.8 | Negative |
| KH002-035 | Manning et al | 706.2 | 1163.4 | 494.8 | 668.0 | 1145.6 | 97.2 | Positive |
| KH002-042 | Manning et al | 243.2 | 951.6 | 181.6 | 326.2 | 342.2 | 156.6 | Negative |
| KH002-043 | Manning et al | 1235.8 | 1224.8 | 53.4 | 133.8 | 155.8 | 52.6 | Positive |
| KH002-052 | Manning et al | 66.8 | 50.4 | 37.6 | 124.2 | 103.6 | 40.6 | Negative |
| KH002-069 | Manning et al | 252.8 | 47.6 | 37.6 | 478.0 | 86.4 | 31.6 | Negative |
| KH003-015 | Manning et al | 996.0 | 1181.4 | 1169.2 | 824.2 | 1239.4 | 211.2 | Positive |
| KH003-030 | Manning et al | 44.8 | 41.0 | 37.2 | 54.2 | 88.4 | 39.2 | Negative |
| KH003-057 | Manning et al | 510.8 | 1198.0 | 1025.0 | 413.2 | 1246.6 | 260.8 | Positive |
| KH003-060 | Manning et al | 631.4 | 928.2 | 48.8 | 117.4 | 111.8 | 27.8 | Positive |
| KH003-062 | Manning et al | 398.0 | 1011.4 | 374.6 | 234.8 | 1100.0 | 77.2 | Positive |
| KH003-063 | Manning et al | 119.4 | 87.0 | 52.6 | 114.6 | 88.2 | 81.4 | Negative |
| KH003-066 | Manning et al | 467.0 | 182.6 | 96.8 | 463.4 | 170.6 | 59.6 | Positive |
| KH003-067 | Manning et al | 864.8 | 1174.4 | 672.8 | 763.2 | 1161.0 | 227.8 | Positive |
| 068 20080731 | Fathi et al | 505.4 | 628.0 | 616.0 | 494.8 | 417.4 | 223.2 | Positive |
| 0698 70080811 | Fathi et al | 506.8 | 664.0 | 543.4 | 342.6 | 505.8 | 212.4 | Positive |
| 0728 70080917 | Fathi et al | 328.2 | 346.2 | 364.8 | 121.4 | 526.2 | 59.6 | Negative |
| 0738 20086009 | Fathi et al | 500.6 | 387.2 | 228.2 | 640.4 | 162.4 | 235.0 | Positive |
| 652_08_7_17 | Fathi et al | 407.4 | 473.8 | 67.2 | 468.4 | 200.2 | 65.2 | Positive |
| 662 08_7_21 | Fathi et al | 697.6 | 691.4 | 588.8 | 406.2 | 549.2 | 265.6 | Positive |
| 672 08_7_25 | Fathi et al | 958.0 | 2604.1 | 1208.8 | 1170.8 | 2382.4 | 564.0 | Positive |
| 685 08_8_1 | Fathi et al | 579.0 | 376.6 | 294.8 | 684.0 | 257.0 | 302.8 | Positive |
| 687 08_8_2 | Fathi et al | 337.6 | 302.0 | 155.4 | 453.4 | 232.4 | 170.6 | Negative |
| 695 08_8_10 | Fathi et al | 510.4 | 523.6 | 437.2 | 643.0 | 315.2 | 233.0 | Positive |
| 699 08_8_12 | Fathi et al | 397.8 | 356.0 | 272.0 | 621.0 | 314.6 | 263.2 | Positive |
| 700 08_8_12 | Fathi et al | 347.8 | 352.4 | 307.6 | 641.6 | 211.6 | 233.6 | Negative |
| 702 08_8_17 | Fathi et al | 430.6 | 423.8 | 138.8 | 470.2 | 120.4 | 182.8 | Positive |
| 703 08_8_17 | Fathi et al | 635.8 | 763.6 | 851.8 | 726.6 | 579.2 | 330.0 | Positive |
| 704 08_8_20 | Fathi et al | 1501.4 | 2428.2 | 1232.0 | 527.8 | 2223.4 | 599.2 | Positive |
| 705 08_8_23 | Fathi et al | 516.6 | 369.4 | 27.2 | 172.8 | 278.0 | 27.4 | Positive |
| 722 08_9_6 | Fathi et al | 714.4 | 541.4 | 154.8 | 714.2 | 539.2 | 119.8 | Positive |
| 731 08_9_23 | Fathi et al | 356.6 | 1398.5 | 468.4 | 163.8 | 1604.9 | 124.0 | Negative |
